# Progression-related loss of stromal Caveolin 1 levels fosters the growth of human PC3 xenografts and mediates radiation resistance

**DOI:** 10.1038/srep41138

**Published:** 2017-01-23

**Authors:** Andrej Panic, Julia Ketteler, Henning Reis, Ali Sak, Carsten Herskind, Patrick Maier, Herbert Rübben, Verena Jendrossek, Diana Klein

**Affiliations:** 1 Institute of Cell Biology (Cancer Research), University of Duisburg-Essen, University Hospital, Virchowstrasse 173, 45122 Essen, Germany; 2 Department of Urology and Urooncology, University of Duisburg-Essen, University Hospital, Essen, Hufelandstr. 55, 45122 Essen, Germany; 3 Institut of Pathology, University of Duisburg-Essen, University Hospital, Hufelandstr. 55, 45122 Essen, Essen, Germany; 4 Department of Radiotherapy, University of Duisburg-Essen, University Hospital, Hufelandstr. 55, 45122 Essen, Germany; 5 Department of Radiation Oncology, University Hospital, Medical Faculty Mannheim, Heidelberg University, Theodor-Kutzer-Ufer 1-3, 68167 Mannheim, Germany

## Abstract

Despite good treatment results in localized prostate tumors, advanced disease stages usually have a pronounced resistance to chemotherapy and radiotherapy. The membrane protein caveolin-1 (Cav1) functions here as an important oncogene. Therefore we examined the impact of stromal Cav1 expression for tumor growth and sensitivity to ionizing radiation (IR). Silencing of Cav1 expression in PC3 cells resulted in increased tumor growth and a reduced growth delay after IR when compared to tumors generated by Cav1-expressing PC3 cells. The increased radiation resistance was associated with increasing amounts of reactive tumor stroma and a Cav1 re-expression in the malignant epithelial cells. Mimicking the human situation these results were confirmed using co-implantation of Cav1-silenced PC3 cells with Cav1-silenced or Cav1-expressing fibroblasts. Immunohistochemically analysis of irradiated tumors as well as human prostate tissue specimen confirmed that alterations in stromal-epithelial Cav1 expressions were accompanied by a more reactive Cav1-reduced tumor stroma after radiation and within advanced prostate cancer tissues which potentially mediates the resistance to radiation treatment. Conclusively, the radiation response of human prostate tumors is critically regulated by Cav1 expression in stromal fibroblasts. Loss of stromal Cav1 expression in advanced tumor stages may thus contribute to resistance of these tumors to radiotherapy.

The clinical relevance of the tumor microenvironment in modulating the response of solid tumors to chemotherapy and radiotherapy has been documented^1^,^2^,^3^,^4^,^5^. Herein, the membrane protein caveolin-1 (Cav1) came into focus as it is overexpressed or mutated in many solid human tumors^6^,^7^,^8^,^9^,^10^,^11^. Although Cav1 acts as tumor suppressor in non-transformed cells, its overexpression has been linked to tumor progression and poor prognosis^12^,^13^,^14^,^15^. As an example, overexpression of Cav1 has been identified as a marker for breast, lung and prostate cancer (PCa) progression that is associated with increased resistance to chemotherapy, metastatic disease and poor prognosis^16^,^17^. Furthermore, patients with advanced PCa had also increased serum levels of Cav1 suggesting a secretion of Cav1 from PCa cells that may contribute to the tumor-promoting effects of Cav1^18^. Interestingly, though levels of Cav1 increased in epithelial cancer cells during PCa progression, Cav1 expression was decreased in the tumor stroma in advanced and metastatic PCa, an effect that was found to be functionally relevant to tumor progression and to correlate with reduced relapse-free survival^10^,^19^. It is assumed that regulated Cav1 expression in the cancer cells is a prerequisite for their hyperproliferative stage and that Cav1 might regulate tumor-promoting epithelial-mesenchymal transition (EMT) of the transformed epithelial cells, tumor angiogenesis and metastasis^20^. Regulation of Cav1 function was further related to signaling by receptor-independent tyrosine kinases (Src, Abl) or oncogenes (c-myc, v-Abl, H-Ras), to the inactivation of tumor suppressor genes (p53), as well as to posttranslational modifications such as phosphorylation or palmitoylation^21^. Altogether these observations demonstrate that in the context of the altered genetic background of transformed cells Cav1 mediates altered cellular functions such as apoptosis resistance and metastasis^22^. Studies in other cancer types further implicated Cav1 as a pro-survival factor mediating resistance e.g. in pancreatic and lymphoblastoid cancer cells to the cytotoxic action of ionizing radiation (IR) *in vitro*. Silencing of Cav1 in pancreatic cancer cell lines resulted in the disruption of its interactions with beta1-integrin and focal adhesion kinase leading to reduced cell adhesion, proliferation and survival after exposure to IR^23^,^24^,^25^. Similarly, Cav1 expression also protected lymphoblastoid TK6 from radiation-induced apoptosis^26^.

For PCa therapy radical prostatectomy, hormone ablation therapy, percutaneous radiotherapy and interstitial radiation methods are available today for treatment of localized stages^27^,^28^,^29^,^30^. Radiotherapy (RT) is also an integral part of treatment protocols for inoperable locally advanced PCa. However, resistance to chemotherapy and RT remains a major obstacle in the successful treatment of high-risk PCa patients. Herein the role of Cav1 for the outcome of RT in the context of tumor-stroma interactions is still largely unknown.

Consistent with earlier findings we recently demonstrated that increased expression of Cav1 in epithelial cancer cells of advanced human PCa tissue specimens was paralleled by a reduction of Cav1 in the tumor stroma which is well known to have a more reactive phenotype in advanced prostate carcinoma^19^,^31^,^32^,^33^. Importantly alterations in stromal Cav1 levels did not include the tumor vasculature because independent of the tumor stages Cav1 was highly expressed in tumor endothelial cells, even in advanced prostate carcinomas. Cav1 may therefore constitute a valuable therapeutic target to overcome therapy resistance by sensitizing both, radioresistant tumor cells and the radioresistant tumor vasculature, to the cytotoxic effects of IR^19^. Nevertheless, the accelerated growth of untreated prostate tumors in the Cav1-deficient background hinted to a potential risk of treatment strategies targeting endothelial Cav1 for radiosensitization in these tumors making careful validation of such treatment strategies with respect to adverse growth promoting effects absolutely necessary^19^.

Therefore it has to be elucidated whether Cav1-dependent resistance-promoting signals from endothelial cells (EC) can be separated from Cav1-dependent stromal signals that restrict tumor growth and may thus allow a safer targeting of Cav-1 mediated radiation resistance. Here we investigated the role of stromal Cav1 for growth- and resistance-promoting tumor-stroma interactions during PCa progression with a focus on the impact of stromal fibroblasts.

## Results

### Reduction of Cav1 levels decreased survival of clonogenic epithelial cells *in vitro*

**Figure 1:**
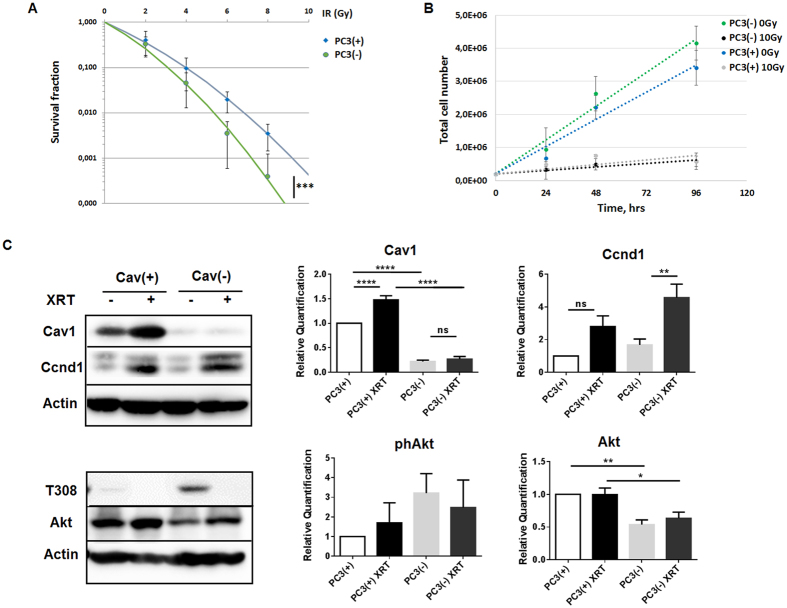
Reduction of Cav1 levels decreased survival of clonogenic epithelial PC3 while proliferation was increased *in vitro*. (**A**) PC3 (shCav1-transfected tumor cells [PC3(−)] as well as PC3 shCtrl control cells [PC3(+)] with normal Cav1 expression) cells were plated for colony formation assay, irradiated with indicated doses (0–8 Gy) and subsequently further incubated for additional 10 days. Data show the surviving fractions from three independent experiments measured in triplicates each (means ± SD). ***P ≤ 0.005 by two-tailed students T-test. (**B**) Cell proliferation was analyzed by cell counting in cultured shCav1-transfected PC3(−) and control-transfected PC3(+) epithelial cells at the indicated time points after irradiation with 10 Gy. Data are shown as means ± SEM of three independent experiments. (**C**) Expression levels of the indicated proteins were analyzed in whole protein lysates of cultured PC3 cells (+/−Cav1) with or without radiation (48 hours after XRT with 10 Gy) using Western blot analysis. Representative blots are shown. For quantification blots were analyzed by densitometry and the respective signal was related to beta-actin (n = 4–5 for each group). For determination of the Akt phosphorylation status the obtained phospho-specific signal was related to the signal of the total protein (phAkt/Akt). P-values were indicated: *P ≤ 0.05, **P ≤ 0.01, ****P ≤ 0.001, by one-way ANOVA followed by post-hoc Tukey test.

To investigate whether reduced Cav1 expressions might alter the radiation response of malignant prostate epithelial cells we performed *in vitro* experiments using the human prostate carcinoma cell line PC3 in combination with shRNA knock-down of Cav1 expression (Fig. 1). Using long-term assays measuring the surviving fraction after irradiation revealed that the number of epithelial PC3 cells able to re-grow and form a colony after irradiation was considerably diminished in shCav1 PC3(−) cells as compared to the shCtrl PC3(+) cells with normal Cav1 expression (Fig. 1A). The reduction of Cav1 levels resulted in a slight but not significant increase in epithelial cell proliferation (Fig. 1B). Radiation further fostered a significant upregulation of Cav1 expression levels in shCtrl PC3(+) but not in shCav1 PC3(−) (Fig. 1C). Expression levels of the proliferation marker cyclin D1 (Ccnd1) were furthermore significantly increased in shCav1 PC3(−) upon radiation. Further examination of the expression levels of the survival protein Akt/ Protein kinase B showed that the more radio-sensitive shCav1 PC3(−) showed significantly decreased expression levels of Akt as compared to Cav1- expressing shCtrl PC3(+). Consequently lowering Cav1 levels specifically in tumor epithelial cells may be suited to increase the efficiency of IR in PCa.

### Single dose irradiation decreased growth of PC3 xenograft tumors more efficiently in Cav1-expressing PC3 tumors which was accompanied by a less reactive tumor stroma

**Figure 2:**
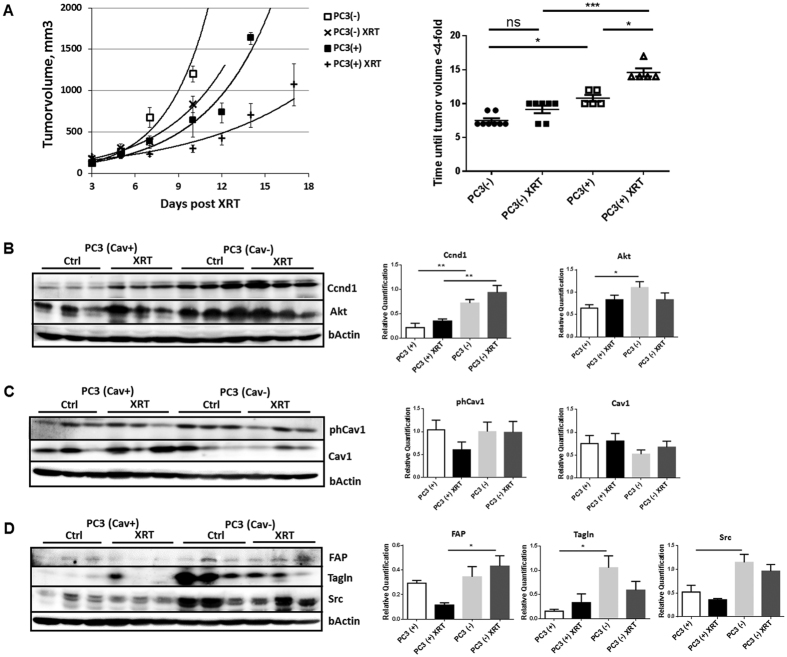
Single dose irradiation (10 Gy) decreased growth of PC3 xenograft tumors more efficiently in Cav1-expressing PC3 tumors which was accompanied by a less reactive tumor stroma. (**A**) PC3 shCav1 tumor cells [PC3(−)] as well as PC3 shCtrl control cells [PC3(+)] with normal Cav1 expression (0.5*10^6^ cells each) were subcutaneously transplanted onto the hind limb of NMRI nude mice. One set of animals from each group received a single radiation dose of 10 Gy to the tumor after manifestation of the tumor at day 3. Tumor volume was determined at indicated time points using a sliding caliper (left diagram). Data are presented as mean ± SEM from 3 independent experiments (25 mice in total: PC3(+) 0 Gy n = 5; PC3(+) 10 Gy n = 5; PC3(−) 0 Gy n = 8; PC3(−) 10 Gy n = 7). Tumor growth and respective computed median growth delay was determined as time (days) until a four-fold tumor volume was reached (right diagram). *p < 0.05, ***p < 0.005 by one-way ANOVA followed by post-hoc Tukey’s test. (**B**–**D**) Expression levels of the indicated proteins were analyzed in whole protein lysates using Western blot analysis. Representative blots are shown. For quantification blots were analyzed by densitometry and the respective signal was related to beta-actin (at least n = 4 for each group). When the phosphorylation status was determined the obtained phospho-specific signal was related to the signal of the total protein (phCav1/Cav1). P-values were indicated: **P ≤ 0.01, ***P ≤ 0.01, by one-way ANOVA followed by post-hoc Tukey test.

To examine the role of epithelial Cav1 in prostate tumor radiosensitivity *in vivo*, we next compared the response to a single high dose irradiation in PC3(−) tumor xenografts to that of Cav1-expressing PC3(+) cells, because the above *in vitro* data suggested that the Cav1-silenced PC3 cells are more sensitive to IR (Fig. 2). For this, subcutaneous PC3 prostate xenografts were implanted onto the hind limb of NMRI nude mice and were irradiated locally with a single dose of 10 Gy when the tumor reached a size of about 100 mm^3^ (around day 3). Tumor growth was determined by measuring the tumor volume 3 times a week (Fig. 2A). PC3(−)-derived tumors showed a significantly increased tumor growth when compared to PC3(+)-derived tumors as demonstrated by the reduced time to reach a four-fold tumor volume (Fig. 2A). Moreover, tumor growth delay after radiation was significantly decreased in shCav1 PC3(−)-derived tumors as demonstrated by the reduced time to reach a four-fold tumor volume (PC3(+) 0 Gy: 10,80 ± 0,49d, n = 5; PC3(+) 10 Gy: 14,60 ± 0,60d, n = 5; PC3(−) 0 Gy: 7,50 ± 0,33d, n = 8; PC3(−) 10 Gy: 9,14 ± 0,55d, n = 7). Because of this discrepancy in radiosensitivity between the *in vitro* and *in vivo* findings we investigated the levels of the proliferation marker Ccnd1 in whole tumor lysates by Western blot analysis (Fig. 2B). In line with the observed *in vitro* findings shCav1 PC3-derived tumors showed significantly higher protein levels of Ccnd1. Ccnd1 expression levels were even significantly increased in irradiated shCav1 PC3(−)-derived tumors as compared to irradiated PC3(+)-derived tumors. We further examined the expression levels of the survival protein Akt (Fig. 2B). Significantly increased expression levels of Akt were detected in PC3(−)-tumors. Surprisingly the Cav1 expression levels were not significantly reduced in tumors grown from Cav1-silenced PC3(−) cells (Fig. 2C). Together with the fact that *in vitro* PC3(−) cells showed an increased proliferation rate and a significantly increased sensitivity to IR we speculate that differences in the stromal compartment between tumors grown from PC3(−) and Cav1-expressing PC3(+) cells may contribute to the observed findings. We therefore investigated the expression levels of the reactive stroma markers fibroblast activating protein (FAP) and transgelin (Tagln), as well as c-Src, a non-receptor tyrosine kinase that regulates a complex signaling network and that has been implicated in both epithelial and stromal mechanisms of disease progression (Fig. 2D). Interestingly higher expression levels of these proteins were detected in PC3(−)-tumors indicating the presence of a more reactive tumor stroma. In particular after irradiation the presence of a more reactive tumor stroma might account for the observed reduced sensitivity to IR *in vivo*.

### Immunohistological analysis of experimental PC3 prostate xenografts confirmed the presence of a more reactive tumor stroma which potentially caused the radiation resistance

**Figure 3:**
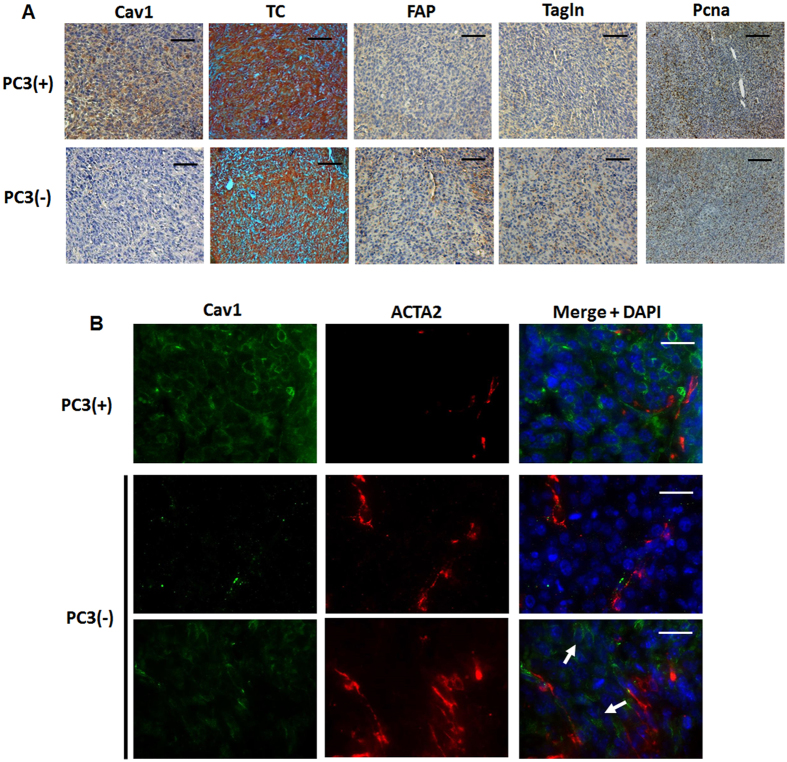
Prostate tumors grown from Cav1-silenced PC3 cells were accompanied by a more reactive tumor stroma. (**A**) Tumors derived from shCav1 PC3 cells [PC3(−)] as well as from PC3 shCtrl control cells [PC3(+)] with normal Cav1 expression were removed when tumor volumes reached a critical size (8–14 days after implantation) and were then subjected to immunohistochemistry with the indicated antibodies. Masson´s Goldner Trichrome (TC) was performed in order to visualize the collagenous stroma. Representative images are shown. Sections were counterstained using hematoxylin. Magnification Cav1, TC, FAP, Tagln 20x; Pcna 10x. (**B**) Subcutaneously grown tumors were further analyzed by immunofluorescence and confocal microscopy. Tumor stroma was stained for smooth muscle actin (ACTA2; red) and Cav1 (green). Arrows point towards Cav1-positive PC3(−) epithelial cells which were supposed to become immunoreactive for Cav1 upon tumor progression. Representative images from at least three independent experiments are shown. Magnification 63x (scale bar 50 μm).

To corroborate the findings of a more reactive tumor stroma in Cav1-silenced PC3(−)-tumors tissues derived from PC3(−) cells as well as from Cav1-expressing PC3(+) cells were subjected to immunohistochemistry for Cav1, FAP and Tagln (Fig. 3A). As expected nearly no epithelial Cav1 expression was detected in tumors derived from Cav1-silenced PC3(−) cells. As compared to Cav1-expressing PC3(+)-tumors, PC3(−)-tumors contain a more collagenous tumor stroma as revealed by the increased blue-green stromal compartment after a Masson’s Goldner Trichrome staining. After radiation there was markedly increased Cav1 expression detectable in these PC3(−)-derived tumors which was paralleled by an increased immunoreactivity to FAP and Tagln in the stromal compartment (Supplemental Figure S1). This confirmed our Western blot results that tumors derived from PC3(−) cells have higher amounts of stromal marker proteins and that in particular after radiation treatment the more radioresistant PC3(−)-tumors are characterized by a more reactive tumor stroma. In line with the observed increase in tumor growth of shCav1 PC3-derived tumors we further detected an increased immunoreactivity to the universal proliferation marker proliferating cell nuclear antigen (Pcna) in these tumors (Fig. 3A). Immunofluorescence analysis further confirmed that the increase in proliferation was mainly caused by the malignant epithelial cells itself as nearly no Pcna-immunoreactivity was detectable in Tagln-positive stromal cells (Supplemental Figure S2). Immunofluorescent analysis of the stromal marker protein smooth muscle actin (ACTA2) together with Cav1 further revealed that in PC3(−)-derived tumors some Cav1 expressing epithelial cells can be detected close to fibroblast-enriched tumor regions, which led us speculate that the recruited stromal cells might mediate partially Cav1 re-expression in the prostate epithelial PC3 cells which because of the phenotype of implanted stable transduced PC3(−) cells must be due to a Cav1 substitution (Fig. 3B, arrows). Radiation further seemed to induce an increase in Cav1 re-expression in tumors of Cav1-silenced PC3(−) cells (Supplemental Figure S1). These results suggested that close to the human situation an increase in epithelial Cav1 (re-) expression together with a more reactive tumor stroma may account for the observed increase of radiation resistance.

**Figure 4:**
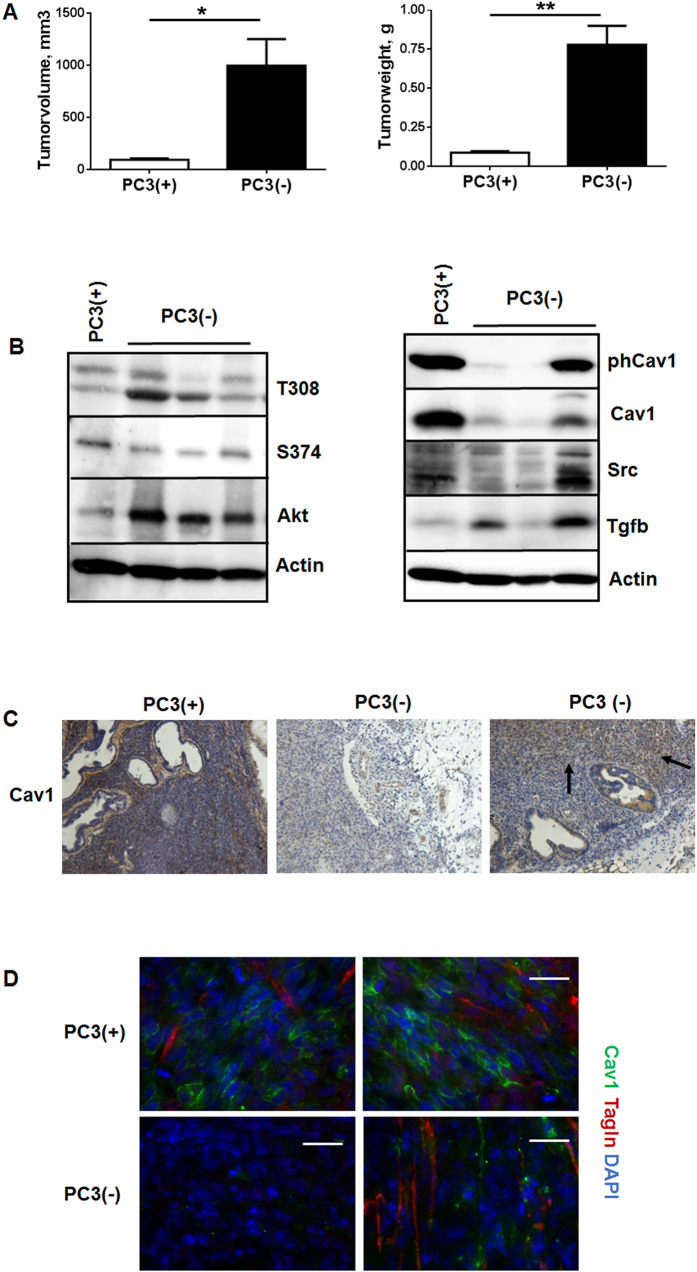
Orthotopic tumors derived from Cav1-silenced PC3(−) cells showed a significantly increased tumor growth and epithelial Cav1 (re-) expressions. (**A**) PC3 (+/−Cav1) cells were injected into the right and left dorsolateral lobe (0.5 × 10^5^ cells per lobe) of the prostate of NMRI nude mice. Tumor weight and volume was determined 14 days after tumor cell implantation. Data are presented as mean ± SEM from 3 independent experiments (16 mice in total: PC3(+) n = 7; PC3(−) n = 9); *P ≤ 0.05, **P ≤ 0.01 by two-tailed t-tests with Welch’s correction. (**B**) Expression levels of the indicated proteins were analyzed in whole protein lysates using Western blot analysis. Representative blots are shown. (**C**) Tumors derived from shCav1 PC3 cells as well as from shCtrl control cells with normal Cav1 expression were subjected to immunohistochemistry with Cav1 antibody. Representative images are shown. Sections were counterstained using hematoxylin. Arrows point towards Cav1-positive epithelial cells within tumors derived from implanted PC3(−) cells. Magnification 20x. (**D**) Orthotopic grown tumors were further analysed by immunofluorescence and confocal microscopy. Tumor stroma was stained for Tagln (red) and Cav1 (green). Representative images from at least three independent experiments are shown. Magnification 63x (scale bar 50 μm).

In order to corroborate these findings and to mimic the human situation more precisely we implanted Cav1-silenced PC3 cells as well as Cav1-expressing control cells directly into the right and left dorsolateral lobe of the prostate of NMRI nude mice (Fig. 4). In line with the results obtained above PC3(−)-derived tumors showed a significantly increased tumor growth when compared to PC3(+)-derived tumors as demonstrated by the significantly increased tumor weight and volume 14 days after tumor cell implantation (Fig. 4A). Akt expression levels were also increased in shCav1 PC3(−)-derived tumors as compared to PC3(+)-derived tumors (Fig. 4B). The Cav1 expression levels were reduced in tumors grown from Cav1-silenced PC3(−) cells but the lower Cav1 expression levels varied between the different shCav1 PC3(−)-derived tumors. Interestingly we found a variable upregulation of the EMT promoting growth factor transforming growth factor beta 1 (TGFb) within these tumors. Also, higher expression levels of the reactive stroma markers FAP and Tagln were detected in PC3(−)-tumors confirming the presence of a more reactive tumor stroma (not shown). Immunohistochemical analysis of Cav1 further showed that in PC3(−)-derived tumors some areas with Cav1-expressing epithelial cells can be detected (Fig. 4C). These Cav1-immunoreactive PC3 cells were again localized within fibroblast-enriched tumor regions (Fig. 4D).

### Reduction of Cav1 levels increased survival of clonogenic fibroblasts *in vitro*

**Figure 5:**
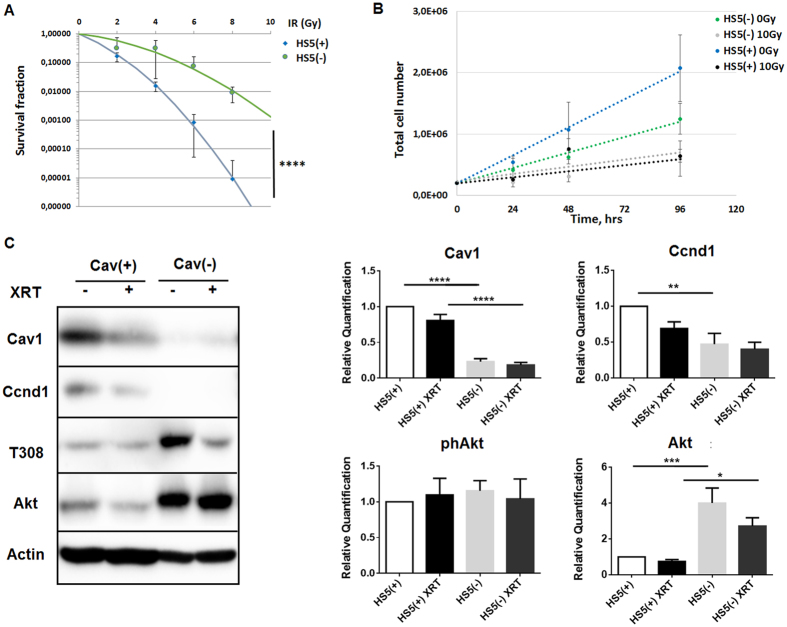
Reduction of Cav1 levels increased survival of clonogenic epithelial PC3 and stromal HS5 cells while proliferation was decreased *in vitro.* (**A**) Lentiviral expression of a Cav1-specific siRNA (shCav1) in stromal HS5 fibroblasts resulted in an efficient and sustained down-regulation of Cav1 expression compared to control-transduced (shCtrl) cells as shown by Western blot analysis. β-actin (bActin) was included as loading control. Representative blots of at least three different experiments are shown. (**A**) HS5 (shCav1-transfected fibroblasts [HS5(−)] as well as shCtrl control cells [HS5(+)] with normal Cav1 expression) cells were plated for colony formation assay, irradiated with indicated doses (0–8 Gy) and subsequently further incubated for additional 10 days. Data show the surviving fractions from three independent experiments measured in triplicates each (means ± SD). ****P ≤ 0.001 by two-tailed students T-test. (**B**) Cell proliferation was analyzed by cell counting in cultured shCav1-transfected HS5(−) and control-transfected HS5(+) fibroblasts cells at the indicated time points after irradiation with 10 Gy. Data are shown as means ± SEM of three independent experiments. (**C**) Expression levels of the indicated proteins were analyzed in whole protein lysates of cultured HS5 cells (+/−Cav1) with or without radiation (48 hours after XRT with 10 Gy) using Western blot analysis. Representative blots are shown. For quantification blots were analyzed by densitometry and the respective signal was related to beta-actin (n = 4–5 for each group). For determination of the Akt phosphorylation status the obtained phospho-specific signal was related to the signal of the total protein (phAkt/Akt). P-values were indicated: *P ≤ 0.05; **P ≤ 0.01; ***P ≤ 0.005 ****P ≤ 0.001; by one-way ANOVA followed by post-hoc Tukey test.

To investigate whether reduced Cav1 expressions might alter the radiation response of stromal fibroblasts we performed *in vitro* experiments using the human fibroblast cell line HS5 as model in combination with shRNA knock-down of Cav1 expression (Fig. 5). According to the experiments with the PC3 cells as described above a long-term assays measuring the surviving fraction after irradiation revealed that the clonogenic survival of Cav1-silenced HS5(−) cells was significantly increased after irradiation as compared to the Cav1-expressing HS5(+) cells (Fig. 5A). Furthermore HS5(−) fibroblasts showed a decreased proliferation rate (Fig. 5B). Radiation further fostered a downregulation of Cav1 expression levels in shCtrl HS5(+) by tendency but not in shCav1 HS5(−) (Fig. 5C). Expression levels of the proliferation marker cyclin D1 (Ccnd1) were furthermore significantly decreased in shCav1 HS5(−). Further examination of the Akt expression levels revealed that the more radio-resistant shCav1 HS5(−) showed significantly increased expression levels of Akt as compared to Cav1-expressing shCtrl HS5(+). Conclusively the impact of the increased survival of Cav1-deficient fibroblasts on the radiation treatment outcome remains to be determined but argues against a general approach for Cav1-silencing in PCa.

### Cav1-deficient stromal fibroblasts mediated radiation resistance

**Figure 6:**
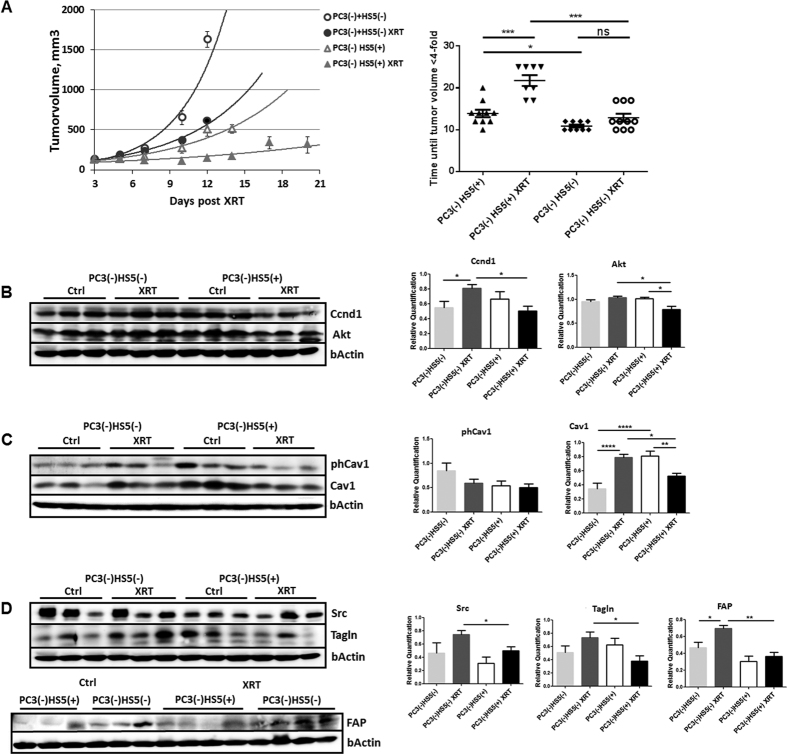
Cav1-deficient stromal fibroblasts mediated radiation resistance. (**A**) Co-implantations of PC3 tumor cells (0.5*10^6^ cells each) after Cav1 silencing [shCav1, PC3(−)] in combination HS5 Cav1-silenced fibroblasts [shCav1, HS5(−)] (0.5*10^6^ cells each) or control fibroblasts [shCtrl, HS5(+)] were performed by subcutaneously transplanted onto the hind limb of NMRI nude mice. One set of animals from each group received a single radiation dose of 10 Gy to the tumor after manifestation of the tumor at day 3. Tumor volume was determined at indicated time points using a sliding caliper (left diagram). Data are presented as mean ± SEM from 3 independent experiments (37 mice in total: PC3(−)HS5(+) 0 Gy n = 10; PC3(−)HS5(+) 10 Gy n = 8; PC3(−)HS5(−) 0 Gy n = 9; PC3(−)HS5(−) 10 Gy n = 10). Tumor growth and respective computed median growth delay was determined as time (days) until a four-fold tumor volume was reached (right diagram). *p < 0.05, ***p < 0.005 by one-way ANOVA followed by post-hoc Tukey’s test. (**B**–**D**) Expression levels of indicated proteins were analyzed in whole protein lysates using Western blot analysis. Representative blots are shown. For quantification blots were analyzed by densitometry and the respective signal was related to beta-actin (at least n = 4 for each group). When the Cav1 phosphorylation status was determined the obtained phospho-specific signal was related to the signal of the total Cav1 protein [phCav1/Cav1]. P-values were indicated: *p < 0.05, **p ≤ 0.01, ***p ≤ 0.005, ****P ≤ 0.001 by one-way ANOVA followed by post-hoc Tukey test.

We then aimed to test whether a more reactive fibroblastic tumor stroma with presumably reduced Cav1 expression accounts for the increased radiation resistance observed in PC3(−) xenograft tumors. To mimic the human situation we performed co-implantations of Cav1-silenced PC3(−) tumor cells in combination with Cav1-silenced or Cav1-expressing HS5 fibroblasts by subcutaneous transplantations onto the hind limb of NMRI nude mice and irradiation treatment after manifestation of the tumor (Fig. 6). In line with the results presented above co-implantation of PC3(−) prostate epithelial cells with HS5 fibroblasts showed that Cav1-silenced HS(−) fibroblasts promoted tumor growth of PC3(−) cells stronger than Cav1-expressing HS5(+) fibroblasts (Fig. 6A). Importantly, the response of PC3(−) xenograft tumors to radiation treatment was significantly less pronounced when they were co-implanted with Cav1-silenced HS5(−) fibroblasts compared to co-implantation with Cav1-expressing HS5(+) fibroblasts as demonstrated by the increased time to reach a four-fold tumor volume (Fig. 4A) (PC3(−)HS5(+) 0 Gy: 13, 9 ± 0, 90 d, n = 10; PC3(−)HS5(+) 10 Gy: 21, 75 ± 1.29 d, n = 8; PC3(−)HS5(−) 0 Gy: 10, 89 ± 0, 35 d, n = 9; PC3(−)HS5(−) 10 Gy: 12, 90 ± 0, 94 d, n = 10).

We investigated again the levels of the proliferation marker Ccnd1 in whole tumor lysates by Western blot analysis (Fig. 6B). Protein levels of Ccnd1 were not altered in HS5(−) co-implanted PC3(−) tumors. After radiation treatment Ccnd1 expression levels were significantly decreased in irradiated shCav1 PC3-derived tumors containing HS5(+) fibroblasts as compared to irradiated shCav1 PC3-derived tumors containing HS5(−) fibroblasts corroborating the data that Cav1-silenced fibroblasts were more radioresistant in respective tumors. We further examined the expression levels of the survival protein Akt and Cav1. Significantly reduced expression levels of Akt were detected in PC3(−)HS5(+)-derived tumors after radiation in line with reduced expression levels of Cav1 (Fig. 6C). Radiation of PC3(−)HS5(−)-derived tumors resulted in significantly upregulated Cav1 levels, which might implicate that epithelial Cav1 re-expression in the prostate epithelial PC3 could contribute to the observed radiation resistance.

Because we speculated that differences in the stromal compartment between PC3 tumors may contribute to the observed findings, we again investigated the expression levels of the reactive stroma markers c-Src, Tagln and FAP as performed above (Fig. 6D). Importantly significant higher expression levels of these proteins were detected in irradiated PC3(−)HS5(−)-derived tumors supporting that a more reactive tumor stroma mediated radiation resistance. We further performed immunohistochemistry analysis to confirm our findings (Supplemental Figure S3). In tumors of Cav1-silenced PC3(−) cells in combination with Cav1-silenced HS5(−) fibroblasts Cav1 immunoreactivity was clearly reduced whereas the reactive stromal markers FAP and Tagln showed a more prominent and intensive staining (Supplemental Figure S3A). And in particular after radiation treatment the more radioresitant PC3(−)HS5(−)-derived tumors displayed an increased immunoreactivity to FAP and Tagln in the stromal compartment, which demonstrated a more reactive tumor stroma after radiation as well as a more prominent staining of epithelial Cav1 (Supplemental Figure S3B). Immunohistochemistry using the Pcna antibody further confirmed the Western blot results because a more intensive staining of the proliferation marker was detected in the more radioresitant PC3(−)HS5(−)-derived tumors and in particular after radiation treatment (Supplemental Figure S3). These results revealed that loss of stromal Cav1 is paralleled by an increase in epithelial Cav1 expression and further suggests again that loss of stromal Cav1 can presumably foster epithelial Cav1 expression and thereby promoting increased of radiation resistance.

### Supernatants derived from Cav1-silenced HS5 fibroblasts fostered radiation resistance of cultured malignant epithelial cells

**Figure 7:**
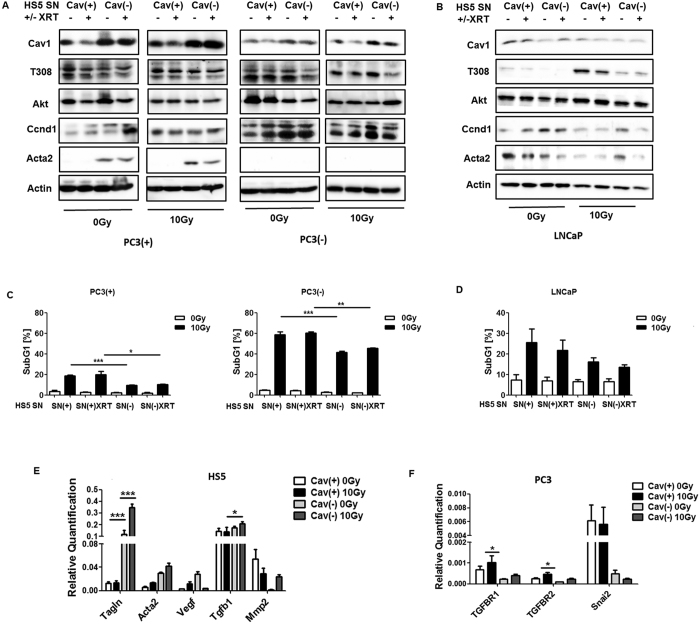
Treatment of cultured PC3 (+/−Cav1) or Cav1-deficient LNCaP malignant epithelial cells with supernatants derived from Cav1-silenced HS5 fibroblasts fostered radiation resistance. (**A**,**B**) PC3 (shCav1-transfected tumor cells [PC3(−)] as well as PC3 shCtrl control cells [PC3(+)] with normal Cav1 expression) and Cav1-deficient LNCaP cells were irradiated with 10 Gy and subsequently treated with supernatants (SN) derived from cultured Cav1-silenced HS5(−) or control transfected Cav1-expressing HS5(+) fibroblasts with or without radiation treatment (+/−XRT with 10 Gy). Western blot analysis of whole protein lysates was performed after 48 hours of treatment using the indicated antibodies. (**C**,**D**) The degree of apoptosis was quantified by measuring the SubG1 fraction 48 hours after radiation by flow cytometry analysis. Therefore, PC3cells (+/−Cav1) and Cav1-deficient LNCaP cells were left non-irradiated (white bars) or irradiated with 10 Gy (black bars) and subsequently treated with SN derived from cultured Cav1-silenced HS5(−) or Cav1-expressing HS5(+) fibroblasts with or without radiation treatment (+/−XRT with 10 Gy). (**E**) Quantitative Real Time RT-PCR (qRT-PCR) analysis of the reactive fibroblasts markers Acta2, Tagln as well as the tumor-promoting factors Vegf (vascular endothelial growth factor), Tgfb and Mmp2 (matrix metalloproteinase 2) were performed in total RNA isolates of Cav1-silenced HS5(−) or control transfected Cav1-expressing HS5(+) fibroblasts with or without radiation treatment (+/−XRT with 10 Gy) and were shown as relative expression to actin (set as 1) at 96 hours post irradiation. Shown are mean values ± SEM from 4 independent samples per group measured in duplicates each. *P ≤ 0.05, ***P ≤ 0.005, by one-way ANOVA followed by post-hoc Tukey’s test. (**F**) Expression levels of the Tgfb receptors TGFBR1 and TGFBR2 as well as the E-cadherin repressing EMT gene Snai2 were analyzed in whole RNA isolates of cultured PC3 cells (+/−Cav1) with or without radiation (48 hours after XRT with 10 Gy) using qRT-PCR analysis. Shown are mean values ± SEM from 4 independent samples per group measured in duplicates each. *P ≤ 0.05 by one-way ANOVA followed by post-hoc Tukey’s test.

Next, to confirm our *in vivo* data mechanistically, *in vitro* analysis of the radiation response of cultured PC3 (+/−Cav1) and Cav1-deficient LNCaP prostate cancer cells were performed in the presence of supernatants (SN) derived from cultured Cav1-silenced HS5(−) or control transduced Cav1-expressing HS5(+) fibroblasts with or without radiation treatment (+/−XRT with 10 Gy) (Fig. 7). Interestingly, SN of Cav1-silenced HS5(−) cells induced a marked upregulation of Cav1 expression levels in cultured PC3(+) cells with and without radiation (Fig. 7A). A similar but less prominent increase in Cav1 was detected in Cav1-silenced PC3(−) cells. No real differences were detected in Akt expression levels upon radiation in combination with HS5 SN treatment. Expression levels of the proliferation marker Ccnd1 were furthermore slightly increased in cultured PC3(+) cells upon treatment with SN of Cav1-silenced HS5(−) cells. The increase of Ccnd1 was more prominent in HS5(−) SN cultured PC3(−) cells. The HS5(−) SN induced upregulation of Cav1 expression levels in cultured PC3(+) cells with and without radiation were paralleled by an upregulation of the mesenchymal marker smooth muscle actin (Acta2). In androgen receptor-expressing and naturally Cav1-deficient LNCaP cells, HS5(+) SN treatment induced a slight upregulation of Cav1 expression levels (Fig. 7B). Again, no real differences were detected in Akt expression levels upon radiation in combination with HS5 SN treatment. A trend of higher Akt phosphorylation levels at threonine 308 (T308) was observed upon HS5(+) SN treatment. Expression levels of Ccnd1 were again slightly increased cultured LNCaP cells upon treatment with SN of Cav1-silenced HS5(−) cells. Interestingly, the HS5(+) SN induced upregulation of Cav1 expression levels were paralleled by an upregulation of Acta2. To further investigate how Cav1 levels might be altered in cultured Cav1-deficient tumor cells Cav1 expression and localization was analyzed in LNCaP cells co-cultured with GFP-expressing (shCtrl)-transfected HS5 fibroblasts by immunofluorescence (Supplemental Figure S4). Direct co-culture of both cell types can lead to an up-regulation of Cav1-immunoreactivity in LNCaP cells suggesting a transfer of Cav1 between the cells (Supplemental Figure S4A). In contrast increased Cav1-immunoreactivity can even be observed in some LNCaP cells upon radiation. Similar findings were observed in Cav1-silenced and GFP-expressing (shCav1-transfected) PC3 cell co-cultures with normal (non-transfected) HS5 fibroblasts (Supplemental Figure S4B). Cav1 secretion was further confirmed by the presence of Cav1 in cell culture supernatants derived from HS5(+/−Cav1) fibroblasts with or without radiation treatment (Supplemental Figure S4C).

The resistance-promoting effect of Cav1-deficient HS5(−) fibroblasts was further analyzed by determining the degree of apoptosis (SubG1 fraction) after radiation in PC3 (+/−Cav1) and Cav1-deficient LNCaP prostate cancer cells in the presence of HS5 SN (Fig. 7C,D). Conformingly SN of Cav1-silenced HS5(−) significantly reduced apoptosis induction in cultured PC3(+) and in the *in vitro* more radiosensitive PC3(−) (Fig. 7C). A similar but less prominently reduced apoptosis rate was detected in LNCaP cells upon radiation and HS5(−) SN treatment (Fig. 7D). Quantitative Real Time RT-PCR analysis of the reactive fibroblasts markers Acta2, Tagln as well as the tumor-promoting factors Vegf (vascular endothelial growth factor), Tgfb and Mmp2 (matrix metalloproteinase 2) in total RNA isolates of HS5 fibroblasts (+/−Cav1 and +/−XRT) further confirmed the more reactive phenotype of Cav1(−) HS5 fibroblasts (Fig. 7E). A general upregulation of the most prominent angiogenic growth factor Vegf in Cav1(−) HS5 fibroblasts was detected, whereas a significant upregulation of the EMT promoting growth factor Tgfb was detected in Cav1(−) HS5 fibroblasts upon radiation. Furthermore reduced Mmp2 mRNA expression levels were detected in HS5(−) fibroblasts which is in line with the findings stated above as reduced expressions of collagenases may also contribute to increased collagen deposition and thus the presence of a more reactive tumor stroma. QRTPCR analysis further confirmed significantly increased mRNA expression levels of the Tgfb receptors TGFBR1 and TGFBR2 as well as the epithelial cadherin repressing EMT gene Snai2 in PC3(+) cells (Fig. 7F). Conclusively the resistance-promoting effect of Cav1-deficient HS5(−) fibroblasts coincided with upregulations of Cav1 expression levels in malignant epithelial cells and further suggests that EMT might contribute to the observed radiation resistance.

### Loss of stromal Cav1 in advanced PCa is accompanied by a more reactive tumor stroma indicating radiation resistance

**Figure 8:**
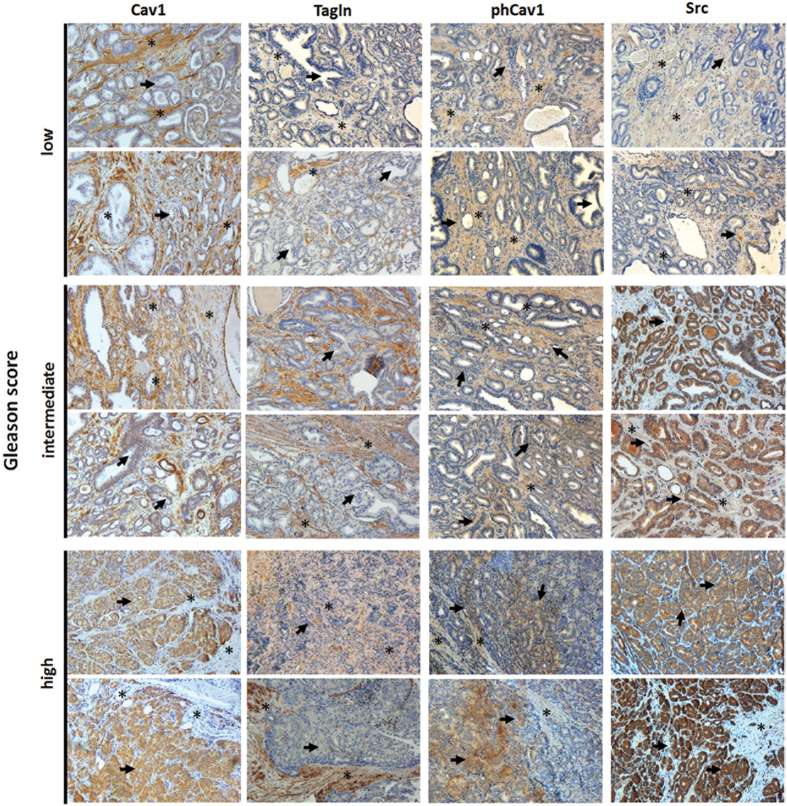
Immunohistological analysis of Cav1 expressions in human prostate tumor tissues. Paraffin-sections of human prostate tumors were stained for the indicated antibodies. Gleason grading scores used to evaluate prognosis of men with prostate cancer were divided into low (1 + 1, 2 + 2), intermediate (3 + 3, 4 + 3) and high scores (4 + 5) according to the sum of the primary and secondary Gleason patterns in whole resection specimens. The observed patterns of the tumor specimen were assigned based on current WHO and updated ISUP criteria: the primary grade - assigned to the dominant pattern of the tumor (has to be greater than 50% of the total pattern seen) as well as a secondary grade - assigned to the next-most frequent pattern (has to be less than 50%, but at least 5%, of the pattern of the total cancer observed). Asterisks mark stromal compartments and bold arrows point to epithelial structures. Sections were counterstained using hematoxylin. Representative images are shown. Magnification 20x.

To confirm that loss of stromal Cav1 is paralleled by a radiation-resistance promoting reactive tumor stroma, human prostate tissue specimens were analyzed for Cav1 and Tagln protein expressions. Therefore, formalin fixed paraffin-embedded tissue slides of human prostate adenocarcinomas with distinct Gleason Scores were immunostained for Cav1, phosphoCav1, Src and Tagln (Fig. 8, Supplemental Figure S5). In line with previous reports we found that benign prostate epithelia were negative for Cav1 but that Cav1 expression in prostate epithelial cells increased with higher Gleason scores, i.e. lower tumor differentiation (Fig. 8, bold arrows). In contrast, stromal cells of tumor samples tended to be less intensively stained or even negative in cases with higher Gleason grade (Fig. 8, asterisks). The increase in stromal Tagln expression in higher Gleason score specimen confirmed the more reactive tumor stroma phenotype. Similar to the Cav1-alterations differences were found for phosphoCav1 and the Cav1 (on Tyr14) phosphorylating kinase Src expressions. Whereas both proteins were more intensively stained in the stromal compartment of tumor specimen with lower Gleason scores, immunoreactivity clearly increased in the malignant epithelial cells of higher Gleason grade tumor specimen. These results indicated that Src-mediated regulations of Cav1 phosphorylation have implications for prostate carcinoma progression and therapy resistance because Src-dependent Cav1 phosphorylation is required for Cav1 signaling prior our suggested potentially stromal-epithelial Cav1 substitution.

## Discussion

Understanding the role of stromal cells in tumor responses to RT and chemotherapy is essential to improve treatment strategies and to reduce the rate of resistant tumors. We recently demonstrated that stromal Cav1 levels in the tumor microvasculature are important to the outcome of RT^19^. Within that previous study murine prostate MPR31–4 tumors grown in Cav1-deficient mice showed significantly increased tumor progression, but upon radiation treatment a more pronounced tumor growth delay, because loss of stromal Cav1 enhanced the sensitivity of microvascular EC to radiation-induced apoptosis^19^. Therefore we concluded that Cav1 might be a promising therapeutic target for combinatorial therapies to counteract radiation resistance of PCa at the level of the tumor vasculature. Accordingly we used cultured PC3 cells and confirmed that loss of Cav1 expression increased sensitivity of PC3 cells to radiation and reduces their clonogenic survival after irradiation, which further supports the idea of Cav1 being a valuable therapeutic target. This is inline with the work from other groups which already demonstrated in other cancer types that Cav1 acts as a pro-survival factor mediating resistance. In particular silencing of Cav1 in pancreatic cancer cell lines and lymphoblastoid cancer cells resulted in reduced cell adhesion, proliferation and survival after exposure to IR^23^,^24^,^25^.

*In vivo*, silencing of Cav1 expression in PC3 cells resulted in an increased tumor growth and reduced growth delay after IR when compared to tumors generated by Cav1-expressing PC3 cells, which was accompanied by increasing amounts of reactive tumor stroma and potentially by a Cav1 re-expression in the malignant epithelial cells. The importance of stromal fibroblasts for the progression and radiation response of prostate tumors was further highlighted after co-implantation of Cav1-silenced PC3 cells with Cav1-proficient or -deficient HS5 fibroblasts as model. We show here for the first time that HS5 fibroblasts with reduced Cav1 levels resulted in increased radioresistance to IR. We further demonstrated for the first time that the decreased radiation-induced growth delay of tumors with co-implanted Cav1-silenced HS5 cells was associated with an increased reactive tumor stroma. Thus, normal Cav1-positive stroma might inhibit tumor progression and improve the efficiency of radiation therapy, whereas a Cav1-dependend transformed and more reactive tumor stroma fosters tumor growth and contributes to therapy resistance. Together with the accelerated growth of untreated prostate tumors when Cav1-silenced fibroblasts were implanted the presented results hint to a potential risk of treatment strategies targeting Cav1 for radiosensitization in these tumors making careful validation of such treatment strategies with respect to adverse growth promoting effects absolutely necessary.

Today it is widely accepted that stroma changes play a functional role during neoplastic transformation and also a key role in cancer cell invasiveness and progression and potentially therapy resistance^34^,^35^. Herein the reactive tumor stroma significantly contributes to therapy resistance at multiple levels^36^,^37^,^38^,^39^,^40^,^41^. In particular therapy-induced DNA damage of the stromal compartment can lead to the activation of secretory programs which in turn influence the growth and survival of tumor cells^42^. On the one hand transcriptional alterations in primary prostate fibroblasts following DNA damage resulted in potential paracrine effects on adjacent tumor cells^43^. Here we now show that tumors from Cav1-silenced PC3 cells were characterized by increasing amounts of reactive tumor stroma and a reduced growth delay after IR and that these tumors displayed significantly higher levels of the survival protein Akt. Similar results were obtained in tumors with co-implanted Cav1-silenced HS5 cells and thus an increased reactive tumor stroma and increased Akt levels. Elevated Akt levels and aberrant activation of PI3K-Akt pathway was already suggested to contribute to increased cell invasiveness and facilitate PCa progression^44^.

As it is not possible to target Cav1 specifically in tumor cells it is important to dissect the molecular details of Cav1-mediateded radiation response modulation. One potential candidate as suggested by the presented results is the non-receptor tyrosine kinase Src. Src has already been implicated in PCa development, progression and metastasis^45^. Accordingly, we show here for the first time that both untreated and irradiated tumors grown from Cav1-silenced PC3 cells after orthotopic or subcutaneous implantation displayed significantly higher Src levels which were in parallel with the observed significantly higher levels of reactive stroma markers. Furthermore, using the co-implantation model, the more radioresitant tumors which contained Cav1-deficient HS5 fibroblasts at the implantation time point, showed significantly higher Src levels in response to IR which again was in parallel with the observed significantly higher levels of reactive stroma markers. Therefore treatment with Src inhibitors might be a potential treatment option for radiosensitizing advanced prostate carcinomas which were characterized by a Cav1-deficient reactive tumor stroma. This is of particular interest since work from another group already demonstrated that the Src inhibitor dasatinib treatment impaired the metastatic phenotypes of the human PCa cell lines PC-3, DU-145, and LNCaP, by significantly reducing migration and invasion^46^. Dasatinib treatment of athymic nude mice resulted in impaired growth of PC3 cell xenograft tumors. Herein, dasatinib also had direct effects on the ability of microvascular EC to form tubes *in vitro* and impaired the ability of PC-3 cells to induce angiogenesis *in vivo*^46^.

In general, Src-mediated Cav1 phosphorylation on Tyr14 has been demonstrated to be essential for Cav1 signaling and Cav1 endocytosis because phosphorylation of Cav1 leads to separation of neighboring negatively-charged N-terminal phospho-tyrosine residues, promoting swelling of caveolae followed by their release from the plasma membrane^47^,^48^. Hereby Src has also been shown to play an important role in PCa development and progression because Src can signal through focal adhesion kinase (FAK) in response to integrin activation, which has been implicated in many aspects of tumor biology, such as cell proliferation, metastasis and angiogenesis^49^,^50^. Therefore Src inhibition represents a valid therapeutic strategy for investigation^46^. Src was further shown to contribute to increases in Cav1 expressions^51^. Furthermore, upregulations of epithelial Cav1 expression followed the induction of EMT and was preceded by increased activation of FAK and Src, two known tyrosine kinases also involved in EMT^51^. Cav1 was already shown to regulate tumor-promoting EMT of transformed epithelial cells and thereby promoting invasive phenotypes e.g. in bladder or gastric cancers^20^,^52^. Herein positive Cav1 expression was significantly correlated with negative E-cadherin expression in malignant epithelial cells^52^. Mechanistically EMT might result from an increased internalization of TGFBRI and Cav1 from lipid rafts which in turn results in an increased TGFb signaling^53^. In line with these findings we show here for the first time that Cav1-deficient fibroblasts foster an upregulation of prostate cancer cell Cav1 expression levels *in vitro* and *in vivo* and furthermore in response to IR treatment and thereby might contribute to the observed radiation resistance.

In a very recently published study DeRita *et al*. demonstrated that Src is packaged into exosomes and released from PCa cells^50^. Furthermore the authors showed that Src- containing exosomes can be isolated in higher amounts from the plasma of prostate tumor-bearing TRAMP mice than wildtype littermates, suggesting that Src signaling network may provide useful biomarkers detectable by liquid biopsy^50^. Thus, Src signaling network may contribute to PCa progression via exosomes. Although the authors did not investigate possible Cav1 presence in this exosomes, it is a quite promising observation since the alterations of Cav1 observed by us and others during PCa progression, maybe either based on direct transfer of Cav1 via those vesicles between malignant epithelial cells and stromal fibroblasts or might be fostered via the transfer of Cav1 regulating components like Src.

In summary, we demonstrate here for the first time that stromal Cav1 is a critical regulator of the sensitivity of PCa to IR in human PCa PC3 xenograft tumors with impact on tumor growth delay after local irradiation. We observed (i) increased Src expression levels in subcutanously or orthotopic PC3(−)-derived tumors as well as in PC3(−)HS5(−)-derived tumors, (ii) increased Src and Cav1 expression levels in the more radioresistant PC3(−)HS5(−)-derived tumors upon radiation, (iii) a potential Cav1 (re)-expression in Cav1-silenced or deficient prostate cancer cells *in vitro* and *in vivo*, (iv) increased Cav1 expression levels of cultured PC3(+) cells upon radiation and in particular after treatment with supernatants derived from the more radioresistant HS5(−), (v) a downregulation of Cav1 in HS5 fibroblasts upon radiation, and (vi) an upregulation of Tgfb expression in HS5(−) fibroblasts as well as increased expression levels of the corresponding Tgfb receptors in Cav1-expressing PC3(+) cells. Finally (vii) similar to the Cav1-alterations, immunoreactivity of phosphoCav1 and the Cav1 phosphorylating kinase Src clearly increased in the malignant epithelial cells of the more radioresistant higher Gleason grade human prostate adenocarcinomas which was paralleled by a more reactive Cav1-deficient tumor stroma, and thus confirms the importance of Src signaling as a potential candidate for future Cav1-mediated radiation response modulation.

Taken together, Cav1 signaling pathways in particular in stromal fibroblasts contribute to this resistance especially to radiation therapy. Our results strongly argue for a Cav1-dependent EMT in prostate cancer progression which is closely linked and thus may account for the observed radiation resistance. However up to now a detailed mechanism how Cav1(−) HS5 fibroblasts foster Cav1 upregulations or (re) expressions in malignant prostate cancer cells and thereby radiation resistance remains elusive.

In particular it is not clear how Cav1–induced (re-)expression fostered by Cav1(−) fibroblasts might promote EMT and potentially thereby radiation therapy resistance as tumors derived Cav1-expressing PC3(+) were more radiosensitive than respective tumors derived from Cav1-silenced PC3(−) cells. However the potential re-distribution of Cav1 we observed *in vivo* when we attempt to mimic the human situation by implanting Cav1(−) PC3 cells (as prostate epithelial cells were Cav1-negative when the cancer develops) coincides with increased radiation resistance. It will be further important to investigate which factors are decisive the critical stromal-epithelial Cav1 alterations during PCa progression and radiation therapy resistance, namely the observed reduction of stromal Cav1 and increased expression of epithelial Cav1. However induced, a ‘simple’ transfer of Cav1 from stromal fibroblasts to the epithelial cells might not be the only mechanism resulting in epithelial Cav1 (re) expressions as Cav1(−) fibroblasts *in vivo* or as supernatants derived from these cells *in vitro* foster radiation resistance of the PCa cells used here. Even more important the amount and phenotype of endogenous or co-implanted fibroblasts at the time point of irradiation remains to be investigated. Here we showed already that Cav1-silenced fibroblasts have a decreased proliferation rate *in vitro*. Even more important *in vivo* an increased immunoreactivity of the proliferation marker Pcna was predominantly detected in the malignant epithelial cells and not within the fibroblasts. Together with the findings that Cav1-silencing induced a more reactive phenotype of HS5 fibroblasts as revealed by the upregulation of the reactive fibroblasts markers Acta2 and Tagln as well as reduced Mmp2 mRNA expression levels *in vitro*, the phenotype and activation state of the fibroblasts seems to be more important than the amount of these cells within a tumor. Further studies are needed to determine this ratio between prostate cancer cells and fibroblasts in the ectopic tumors at the time of irradiation and how the observed fibroblast activation state than correlates with radiation resistance.

In future studies we finally aim to specify the role of Cav1 alterations potentially induced by the Cav1-deficient and more reactive stroma for the radiosensitivity of PCa on molecular level, in particular to identify Cav1-dependent fibroblastic secreted factors which potentially foster the observed Cav1 re-expression in the malignant epithelial cells. This is a necessary step to develop pharmacological strategies to reduce Cav1 expression or inhibit resistance-promoting Cav1-dependent signals, respectively.

## Methods

### Reagents and antibodies

Antibodies against alpha smooth muscle actin (ACTA2), Cav1 were from Santa Cruz (Santa Cruz, CA), against total AKT, phospho-Cav1, Ccdn1 and Src were from Cell Signaling Technology (Danvers, MA, Germany), against Tagln from Proteintech (Chicago, IL), against FAP from Abcam (Cambridge, MA), against Pcna from GeneTex (Irvine, CA) and against beta actin (clone AC-74, A2228) from Sigma-Aldrich (St. Louis, MO).

### Human tumor tissue

Tissues from human prostate carcinomas were obtained during surgery according to local ethical and biohazard regulations. All experiments were performed in strict accordance with local guidelines and regulations. Resected tissue specimens were processed for pathological diagnostic routine in agreement with institutional standards and diagnoses were made based on current WHO and updated ISUP criteria^54^. All studies including human tissue samples were approved by the local ethics committee (Ethik-Kommission) of the University Hospital Essen (Nr. 10–4363). Informed consent (written form) from each patient was obtained. Human tissue samples were analyzed anonymously.

### Cav1 silencing

The human prostate epithelial cell lines PC3, LNCaP and the fibroblast cell line HS5 were from ATCC (Manassas, VA). Levels of Cav1 mRNA level were down-regulated by shRNA technology^19^,^26^. Therefore, a lentivirus based pLentilox3.7 vector for mammalian expression was used as previously described^19^. Within 2–5 days after transduction eGFP-positive cells were sorted in a FACS Vantage cell sorter (BD Biosciences, Heidelberg, Germany).

### Cell viability assay

The number of living cells was determined upon staining of the cells with the vital dye trypan blue. For this, cells were harvested with Trypsin-EDTA, re-suspended in fresh medium, diluted with trypan blue, and counted employing a Neubauer chamber.

### Colony formation assay

For this long-term assay, 200–1600 cells/well were plated in 6-well plates. Radiation with indicated doses was performed using the Isovolt-320-X-ray machine (Seifert-Pantak) at 320 kV, 10 mA with a 1.65 mm aluminum filter and a distance of about 500 mm to the object being irradiated^19^. The X-ray tube operated at 90 kV (~45 keV X-rays) and the dose rate was about 3 Gy/min^55^. Plates were incubated for a total of 10 days to allow growth of single colonies. Cells were then fixed in 3.7% formaldehyde and 70% ethanol and subsequently stained with 0.05% Coomassie Brilliant Blue. Colonies (≥50 cells/colony) were counted under the microscope at fivefold magnification.

### Conditioned Media

Cav1-specific shRNA (shCav1) as well as control-transduced (shCtrl) HS5 cells were cultured in normal growth media until confluence. Cells were left non-irradiated or irradiated with 10 Gy, media were replaced and cells were cultured in the presence of 0.5% fetal bovine serum for 24 hours before collection of media. Control media were generated by incubating the same medium (containing 0.5% fetal bovine serum) without cells. Conditioned media were used as 1/1 mixture with normal growth medium^55^.

### Flow cytometry analyses

For quantification of apoptotic DNA-fragmentation (sub-G1 population), cells were incubated for 15–30 min with a staining solution containing 0.1% (w/v) sodium citrate, 50 μg/ml PI, and 0.05% (v/v) Triton X-100 (v/v) and subsequently analyzed by flow cytometry (FACS Calibur, Becton Dickinson, Heidelberg, Germany; FL-2)^19^.

### Mouse tumor model

Mouse xenograft tumors were generated by subcutaneous injection of 0.5 × 10^6^ cells PC3 (+/−Cav1) either alone or mixed with 0.5 × 10^6^ cells HS5 (+/−Cav1) cells onto the hind limb of the mice (total volume 50 μl) as previously described^19^. Up to 20 animals of each experimental group received a single subcutaneous injection of 0.5 × 10^6^ viable cells. For radiation therapy mice were anesthetized (2% isoflurane) and tumors were exposed to a single dose of 10 Gy ±5% in 5 mm tissue depth (~1.53 Gy/min, 300 kV, filter: 0.5 mm Cu, 10 mA, focus distance: 60 cm) using a collimated beam with a XStrahl RS 320 cabinet irradiator (XStrahl Limited, Camberly, Surrey, Great Britain)^56^. For intraprostatic implantation, mice were anesthetized with ketamine (50 mg/kg) and xylazine (10 mg/kg). A low abdominal transverse incision was made and PC3 (+/−Cav1) cells (1 × 10^5^ in 50 μl of PBS) were injected into the right and left dorsolateral lobe (25 μl per lobe) of the prostate, and the wound was closed with surgical clips. Carpofen (50 μg/10 g bodyweight) was injected subcutaneously as analgesic. Mouse experiments were carried out in strict accordance with the recommendations of the Guide for the Care and Use of Laboratory Animals of the German Government and they were approved by the Committee on the Ethics of Animal Experiments of the responsible authorities [Landesamt für Natur, Umwelt und Verbraucherschutz (LANUV), Regierungspräsidium Düsseldorf Az. 8.87–50.10.37.09.187; Az. 8.87–51.04.20.09.390].

### Immunohistochemistry and immunofluorescence

Paraffin embedded tissue sections were hydrated using a descending alcohol series, incubated for 10–20 min in target retrieval solution (Dako) and incubated with blocking solution (2% FCS/PBS). After permeabilisation, sections were incubated with primary antibodies over night at 4 °C. Antigen was detected with a peroxidase-conjugated secondary antibody (1/250) and DAB staining (Dako). Nuclei were counterstained using hematoxylin. Masson´s Goldner Trichrome (TC) (Carl Roth Karlsruhe, Germany) for histological evaluation of connective tissue was performed according to the manufactures instruction. For immunofluorescence analysis, antigen was detected with an anti-rabbit-Alexa488 and anti-rat-Alexa555-conjugated secondary antibody (1/500). Hoechst 33342 (Invitrogen, Karlsruhe, Germany) was used for nuclei staining. Specimens were analyzed by confocal microscopy.

### Western blot

Whole cell lysates were generated by scraping cells into ice-cold RIPA-P buffer (150 mmol/L NaCl, 1% NP40, 0.5% sodium-desoxycholate, 0.1% sodium-dodecylsulfate, 50 mmol/L Tris/HCL pH 8, 10 mmol/L NaF, 1 mmol/L Na_3_VO_4_), supplemented with complete Protease-Inhibitor-Cocktail (Roche) and performing 2–3 freeze-thaw cycles. Protein samples (50–100 μg total protein) were subjected to SDS-PAGE electrophoresis and Western blots were done as previously described using the indicated antibodies^57^.

### Statistical Analysis

If not otherwise indicated, data were obtained from 3 independent experiments with at least 3 mice each. Statistical significance was evaluated by 1-way ANOVA followed by Tukey’s or Bonferroni multiple comparisons post-hoc test and set at the level of P ≤ 0.05. Data analysis was performed with Prism 5.0 software (GraphPad, La Jolla, California).

## Additional Information

**How to cite this article:** Panic, A. *et al*. Progression-related loss of stromal Caveolin 1 levels fosters the growth of human PC3 xenografts and mediates radiation resistance. *Sci. Rep.* **7**, 41138; doi: 10.1038/srep41138 (2017).

**Publisher's note:** Springer Nature remains neutral with regard to jurisdictional claims in published maps and institutional affiliations.

## Supplementary Material

Supplementary Figures

## References

[b1] Singh S. R., Rameshwar P. & Siegel P. Targeting tumor microenvironment in cancer therapy. Cancer letters, doi: 10.1016/j.canlet.2016.04.009 (2016). 27060765

[b2] Sun Y. Tumor microenvironment and cancer therapy resistance. Cancer letters, doi: 10.1016/j.canlet.2015.07.044 (2015). 26272180

[b3] Cheng C. J. . MicroRNA silencing for cancer therapy targeted to the tumour microenvironment. Nature 518, 107–110, doi: 10.1038/nature13905 (2015). 25409146 PMC4367962

[b4] Masuda S. & Izpisua Belmonte J. C. The microenvironment and resistance to personalized cancer therapy. Nature reviews. Clinical oncology 10, doi: 10.1038/nrclinonc.2012.127-c1 (2013). 23247379

[b5] Swartz M. A. . Tumor microenvironment complexity: emerging roles in cancer therapy. Cancer research 72, 2473–2480, doi: 10.1158/0008-5472.CAN-12-0122 (2012). 22414581 PMC3653596

[b6] Yang H. . Mechanosensitive caveolin-1 activation-induced PI3K/Akt/mTOR signaling pathway promotes breast cancer motility, invadopodia formation and metastasis *in vivo*. Oncotarget 7, 16227–16247, doi: 10.18632/oncotarget.7583 (2016). 26919102 PMC4941310

[b7] El-Gendi S. M., Mostafa M. F. & El-Gendi A. M. Stromal caveolin-1 expression in breast carcinoma. Correlation with early tumor recurrence and clinical outcome. Pathology oncology research: POR 18, 459–469, doi: 10.1007/s12253-011-9469-5 (2012). 22057638

[b8] Koo J. S., Park S., Kim S. I., Lee S. & Park B. W. The impact of caveolin protein expression in tumor stroma on prognosis of breast cancer. Tumour biology: the journal of the International Society for Oncodevelopmental Biology and Medicine 32, 787–799, doi: 10.1007/s13277-011-0181-6 (2011). 21584795

[b9] Nassar Z. D., Hill M. M., Parton R. G. & Parat M. O. Caveola-forming proteins caveolin-1 and PTRF in prostate cancer. Nature reviews. Urology 10, 529–536, doi: 10.1038/nrurol.2013.168 (2013). 23938946

[b10] Ayala G. . Loss of caveolin-1 in prostate cancer stroma correlates with reduced relapse-free survival and is functionally relevant to tumour progression. The Journal of pathology 231, 77–87, doi: 10.1002/path.4217 (2013). 23729330 PMC3978784

[b11] Freeman M. R., Yang W. & Di Vizio D. Caveolin-1 and prostate cancer progression. Advances in experimental medicine and biology 729, 95–110, doi: 10.1007/978-1-4614-1222-9_7 (2012). 22411316

[b12] Shan T. . Loss of stromal caveolin-1 expression: a novel tumor microenvironment biomarker that can predict poor clinical outcomes for pancreatic cancer. PloS one 9, e97239, doi: 10.1371/journal.pone.0097239 (2014). 24949874 PMC4064978

[b13] Gumulec J. . Caveolin-1 as a potential high-risk prostate cancer biomarker. Oncology reports 27, 831–841, doi: 10.3892/or.2011.1587 (2012). 22159333

[b14] Tahir S. A. . Development of an immunoassay for serum caveolin-1: a novel biomarker for prostate cancer. Clinical cancer research: an official journal of the American Association for Cancer Research 9, 3653–3659 (2003). 14506154

[b15] Thompson T. C. . The role of caveolin-1 in prostate cancer: clinical implications. Prostate cancer and prostatic diseases 13, 6–11, doi: 10.1038/pcan.2009.29 (2010). 19581923 PMC2887695

[b16] Sotgia F. . Caveolin-1 and cancer metabolism in the tumor microenvironment: markers, models, and mechanisms. Annual review of pathology 7, 423–467, doi: 10.1146/annurev-pathol-011811-120856 (2012). 22077552

[b17] Goetz J. G., Lajoie P., Wiseman S. M. & Nabi I. R. Caveolin-1 in tumor progression: the good, the bad and the ugly. Cancer metastasis reviews 27, 715–735, doi: 10.1007/s10555-008-9160-9 (2008). 18506396

[b18] Tahir S. A. . Serum caveolin-1, a biomarker of drug response and therapeutic target in prostate cancer models. Cancer biology & therapy 14, 117–126, doi: 10.4161/cbt.22633 (2013). 23114714 PMC3571993

[b19] Klein D. . Endothelial Caveolin-1 regulates the radiation response of epithelial prostate tumors. Oncogenesis 4, e148, doi: 10.1038/oncsis.2015.9 (2015). 25985209 PMC4450264

[b20] Kannan A. . Caveolin-1 promotes gastric cancer progression by up-regulating epithelial to mesenchymal transition by crosstalk of signalling mechanisms under hypoxic condition. Eur J Cancer 50, 204–215, doi: 10.1016/j.ejca.2013.08.016 (2014). 24070739

[b21] Williams T. M. & Lisanti M. P. Caveolin-1 in oncogenic transformation, cancer, and metastasis. American journal of physiology. Cell physiology 288, C494–506, doi: 10.1152/ajpcell.00458.2004 (2005). 15692148

[b22] Quest A. F., Gutierrez-Pajares J. L. & Torres V. A. Caveolin-1: an ambiguous partner in cell signalling and cancer. Journal of cellular and molecular medicine 12, 1130–1150, doi: 10.1111/j.1582-4934.2008.00331.x (2008). 18400052 PMC3865655

[b23] Cordes N. . Human pancreatic tumor cells are sensitized to ionizing radiation by knockdown of caveolin-1. Oncogene 26, 6851–6862, doi: 10.1038/sj.onc.1210498 (2007). 17471232

[b24] Hehlgans S. & Cordes N. Caveolin-1: an essential modulator of cancer cell radio-and chemoresistance. American journal of cancer research 1, 521–530 (2011). 21984970 PMC3186050

[b25] Hehlgans S. . Caveolin-1 mediated radioresistance of 3D grown pancreatic cancer cells. Radiotherapy and oncology: journal of the European Society for Therapeutic Radiology and Oncology 92, 362–370, doi: 10.1016/j.radonc.2009.07.004 (2009). 19665245

[b26] Barzan D., Maier P., Zeller W. J., Wenz F. & Herskind C. Overexpression of caveolin-1 in lymphoblastoid TK6 cells enhances proliferation after irradiation with clinically relevant doses. Strahlentherapie und Onkologie: Organ der Deutschen Rontgengesellschaft … [et al] 186, 99–106, doi: 10.1007/s00066-010-2029-1 (2010). 20127227

[b27] Joniau S. & Van Poppel H. Localized prostate cancer: can we better define who is at risk of unfavourable outcome? BJU international 101 Suppl 2, 5–10 (2008). 10.1111/j.1464-410X.2007.07488.x 18307686

[b28] Ganswindt U. . Combination of celecoxib with percutaneous radiotherapy in patients with localised prostate cancer - a phase I study. Radiat Oncol 1, 9, doi: 10.1186/1748-717X-1-9 (2006). 16722607 PMC1464385

[b29] Kawamorita N. . Radical prostatectomy for high-risk prostate cancer: biochemical outcome. International journal of urology: official journal of the Japanese Urological Association 16, 733–738, doi: 10.1111/j.1442-2042.2009.02352.x (2009). 19674167

[b30] Ploussard G. . Impact of positive surgical margins on prostate-specific antigen failure after radical prostatectomy in adjuvant treatment-naive patients. BJU international 107, 1748–1754, doi: 10.1111/j.1464-410X.2010.09728.x (2011). 20883488

[b31] Pascal L. E. . Gene expression down-regulation in CD90^+^ prostate tumor-associated stromal cells involves potential organ-specific genes. BMC cancer 9, 317, doi: 10.1186/1471-2407-9-317 (2009). 19737398 PMC2745432

[b32] Orr B. . Identification of stromally expressed molecules in the prostate by tag-profiling of cancer-associated fibroblasts, normal fibroblasts and fetal prostate. Oncogene 31, 1130–1142, doi: 10.1038/onc.2011.312 (2012). 21804603 PMC3307063

[b33] Dakhova O. . Global gene expression analysis of reactive stroma in prostate cancer. Clinical cancer research: an official journal of the American Association for Cancer Research 15, 3979–3989, doi: 10.1158/1078-0432.CCR-08-1899 (2009). 19509179 PMC2734921

[b34] Hanahan D. & Weinberg R. A. The hallmarks of cancer. Cell 100, 57–70 (2000). 10647931 10.1016/s0092-8674(00)81683-9

[b35] Hanahan D. & Weinberg R. A. Hallmarks of cancer: the next generation. Cell 144, 646–674, doi: 10.1016/j.cell.2011.02.013 (2011). 21376230

[b36] Bhowmick N. A., Neilson E. G. & Moses H. L. Stromal fibroblasts in cancer initiation and progression. Nature 432, 332–337, doi: 10.1038/nature03096 (2004). 15549095 PMC3050735

[b37] Schlomm T. . Molecular cancer phenotype in normal prostate tissue. European urology 55, 885–890, doi: 10.1016/j.eururo.2008.04.105 (2009). 18501497

[b38] Ellem S. J., De-Juan-Pardo E. M. & Risbridger G. P. *In vitro* modeling of the prostate cancer microenvironment. Advanced drug delivery reviews, doi: 10.1016/j.addr.2014.04.008 (2014). 24816064

[b39] Dakhova O., Rowley D. & Ittmann M. Genes upregulated in prostate cancer reactive stroma promote prostate cancer progression *in vivo*. Clinical cancer research: an official journal of the American Association for Cancer Research 20, 100–109, doi: 10.1158/1078-0432.CCR-13-1184 (2014). 24150235 PMC3947312

[b40] Barron D. A. & Rowley D. R. The reactive stroma microenvironment and prostate cancer progression. Endocrine-related cancer 19, R187–204, doi: 10.1530/ERC-12-0085 (2012). 22930558 PMC3716392

[b41] Huber R. M. . DNA damage induces GDNF secretion in the tumor microenvironment with paracrine effects promoting prostate cancer treatment resistance. Oncotarget 6, 2134–2147, doi: 10.18632/oncotarget.3040 (2015). 25575823 PMC4385841

[b42] Gilbert L. A. & Hemann M. T. DNA damage-mediated induction of a chemoresistant niche. Cell 143, 355–366, doi: 10.1016/j.cell.2010.09.043 (2010). 21029859 PMC2972353

[b43] Sun Y. . Treatment-induced damage to the tumor microenvironment promotes prostate cancer therapy resistance through WNT16B. Nature medicine 18, 1359–1368, doi: 10.1038/nm.2890 (2012). PMC3677971 22863786

[b44] Shukla S. . Activation of PI3K-Akt signaling pathway promotes prostate cancer cell invasion. International journal of cancer. Journal international du cancer 121, 1424–1432, doi: 10.1002/ijc.22862 (2007). 17551921

[b45] Edwards J. Src kinase inhibitors: an emerging therapeutic treatment option for prostate cancer. Expert opinion on investigational drugs 19, 605–614, doi: 10.1517/13543781003789388 (2010). 20367532

[b46] Rice L., Lepler S., Pampo C. & Siemann D. W. Impact of the SRC inhibitor dasatinib on the metastatic phenotype of human prostate cancer cells. Clinical & experimental metastasis 29, 133–142, doi: 10.1007/s10585-011-9436-2 (2012). 22130962

[b47] Zimnicka A. M. . Src-dependent phosphorylation of caveolin-1 Tyr14 promotes swelling and release of caveolae. Molecular biology of the cell, doi: 10.1091/mbc.E15-11-0756 (2016). PMC4927282 27170175

[b48] Nethe M. & Hordijk P. L. A model for phospho-caveolin-1-driven turnover of focal adhesions. Cell adhesion & migration 5, 59–64 (2011). 20948305 10.4161/cam.5.1.13702 PMC3038100

[b49] Desgrosellier J. S. & Cheresh D. A. Integrins in cancer: biological implications and therapeutic opportunities. Nature reviews. Cancer 10, 9–22, doi: 10.1038/nrc2748 (2010). 20029421 PMC4383089

[b50] DeRita R. M. . c-Src, Insulin-Like Growth Factor I Receptor, G-Protein-Coupled Receptor Kinases and Focal Adhesion Kinase Are Enriched into Prostate Cancer Cell Exosomes. Journal of cellular biochemistry, doi: 10.1002/jcb.25611 (2016). PMC5552241 27232975

[b51] Bailey K. M. & Liu J. Caveolin-1 up-regulation during epithelial to mesenchymal transition is mediated by focal adhesion kinase. The Journal of biological chemistry 283, 13714–13724, doi: 10.1074/jbc.M709329200 (2008). 18332144 PMC2376249

[b52] Liang W. . CAV-1 contributes to bladder cancer progression by inducing epithelial-to-mesenchymal transition. Urologic oncology 32, 855–863, doi: 10.1016/j.urolonc.2014.01.005 (2014). 24968949

[b53] Hwangbo C. . Syntenin regulates TGF-beta1-induced Smad activation and the epithelial-to-mesenchymal transition by inhibiting caveolin-mediated TGF-beta type I receptor internalization. Oncogene 35, 389–401, doi: 10.1038/onc.2015.100 (2016). 25893292

[b54] Epstein J. I., Allsbrook W. C. Jr., Amin M. B. & Egevad L. L. The 2005 International Society of Urological Pathology (ISUP) Consensus Conference on Gleason Grading of Prostatic Carcinoma. The American journal of surgical pathology 29, 1228–1242 (2005). 16096414 10.1097/01.pas.0000173646.99337.b1

[b55] Klein D. . Mesenchymal stem cell therapy protects lungs from radiation-induced endothelial cell loss by restoring superoxide dismutase 1 expression. Antioxidants & redox signaling, doi: 10.1089/ars.2016.6748 (2016). PMC5393411 27572073

[b56] Matschke J. . Targeted Inhibition of Glutamine-Dependent Glutathione Metabolism Overcomes Death Resistance Induced by Chronic Cycling Hypoxia. Antioxidants & redox signaling, doi: 10.1089/ars.2015.6589 (2016). 27021152

[b57] Klein D. . Wnt2 acts as a cell type-specific, autocrine growth factor in rat hepatic sinusoidal endothelial cells cross-stimulating the VEGF pathway. Hepatology 47, 1018–1031, doi: 10.1002/hep.22084 (2008). 18302287

