# Supplementary material for: Progression-related loss of stromal Caveolin 1 levels fosters the growth of human PC3 xenografts and mediates radiation resistance
Source: Sci Rep. 2017 Jan 23;7:41138. doi: 10.1038/srep41138 (PMC5255553; doi:10.1038/srep41138)
Supplement: Supplementary Figures [file srep41138-s1.pdf]

Supplementary Information

Progression-related loss of stromal Caveolin 1 levels fosters the growth of human PC3 xenografts and mediates radiation resistance

Andrej Panic[1,2,#], Julia Ketteler[1,#], Henning Reis[3], Ali Sak[4], Carsten Herskind[5], Patrick Maier[5], Herbert Rübben[2], Verena Jendrossek[1,*] and Diana Klein[1,*]

[1]Institute of Cell Biology (Cancer Research), University of Duisburg-Essen, University Hospital, Virchowstrasse 173, 45122 Essen, Germany.
[2]Department of Urology and Urooncology, University of Duisburg-Essen, University Hospital, Essen, Hufelandstr. 55, 45122 Essen, Germany
[3]Institut of Pathology, University of Duisburg-Essen, University Hospital, Hufelandstr. 55, 45122 Essen, Essen, Germany.
[4]Department of Radiotherapy, University of Duisburg-Essen, University Hospital, Hufelandstr. 55, 45122 Essen, Germany
[5]Department of Radiation Oncology, University Hospital, Medical Faculty Mannheim, Heidelberg University, Theodor-Kutzer-Ufer 1-3, 68167 Mannheim, Germany

[#] equal contribution
* shared senior authorship

Supplementary Figures

Supplementary Figure S1

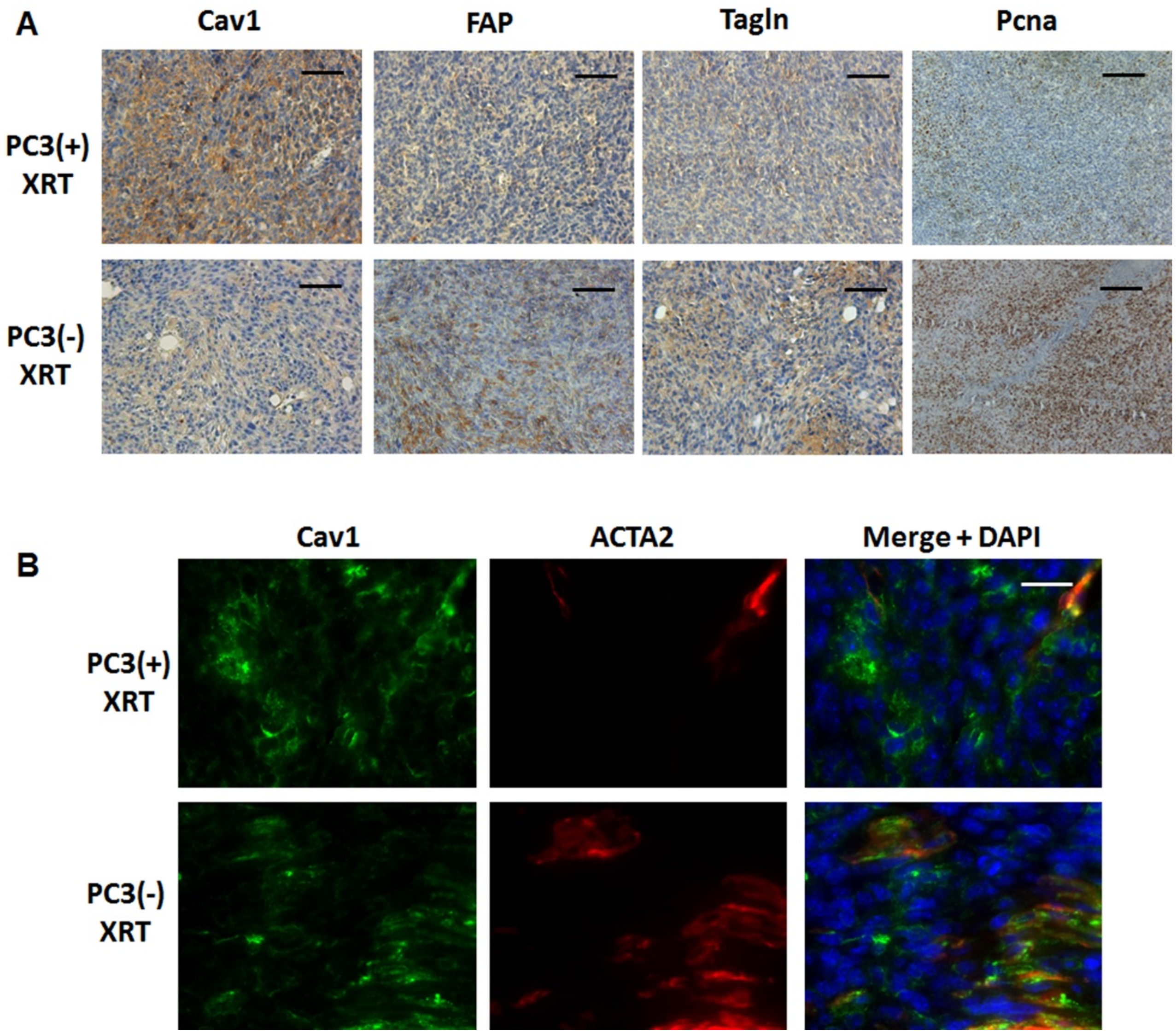

Supplementary Figure S1 | Radiation treatment of prostate tumors grown from Cav1-silenced PC3 cells were accompanied by a more reactive tumor stroma. (A) Tumors derived from PC3(-) as well as from PC3(+) control cells with normal Cav1 expression with radiation treatment (10Gy) were removed when tumor volumes reached a critical size (15-20 days after tumor irradiation) and were then subjected to immunohistochemistry with the indicated antibodies. Representative images are shown. Sections were counterstained using hematoxylin. Magnification Cav1, FAP, Tagln 20x; Pcna 10x. (B) Subcutaneously grown tumors were further analysed by immunofluorescence and confocal microscopy. Tumor stroma was stained for smooth muscle actin (ACTA2; red) and Cav1 (green). Representative images from at least three independent experiments are shown. Magnification 63x (scale bar 50µm).

Supplementary Figure S2

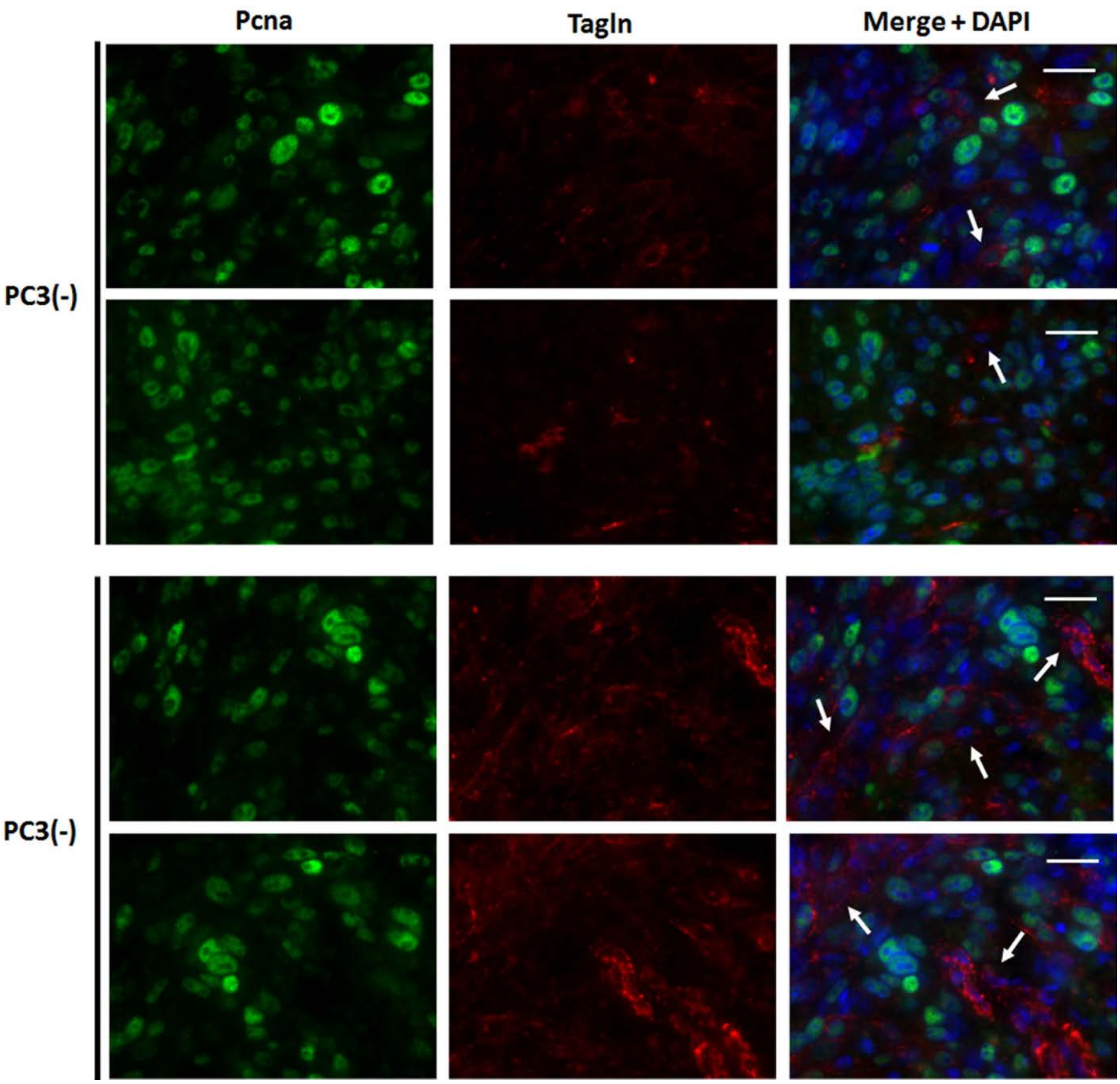

Supplementary Figure S2 | Proliferation was mainly restricted to the malignant epithelial cells. Tumors derived from PC3(-) as well as from PC3(+) control cells with normal Cav1 expression were removed when tumor volumes reached a critical size (8-14 days after implantation) and were analysed by immunofluorescence and confocal microscopy. Sections were stained for the proliferation marker Pcna (green) and tumor stroma was visualized by Tagln-immunoreactivity (red). Representative images are shown. Arrows point towards Pcna-negative stromal cells which were immunoreactive for Tagln. Magnification 63x (scale bar 25µm).

Supplementary Figure S3

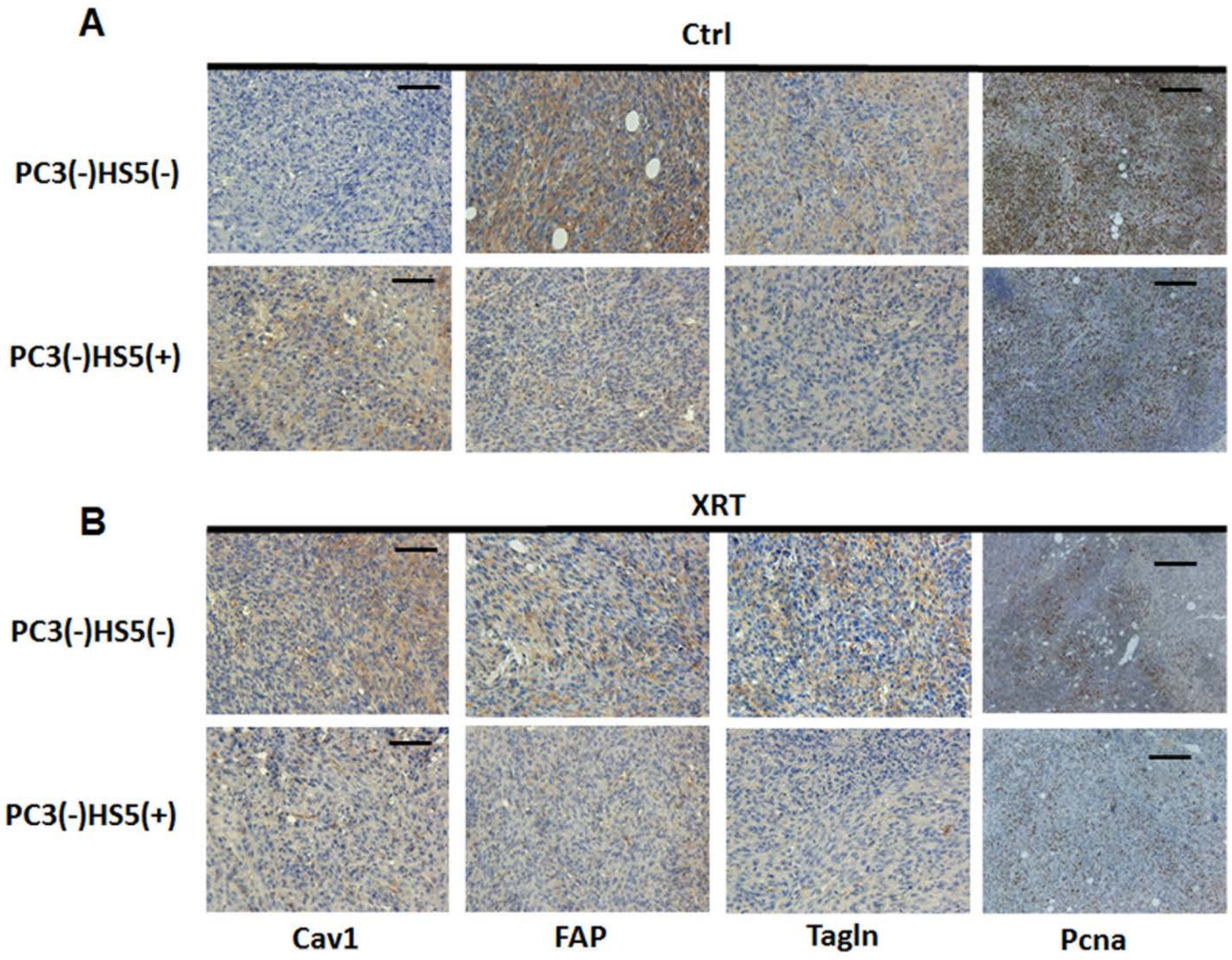

Supplementary Figure S3 | Radiation-induced Cav1 alterations mimicked the human situation: a more reactive tumor stroma potentially supports the resistance to radiation treatment. (A) Tumors derived from shCav1 PC3(-) cells in combination with Cav1-silenced HS5(-) fibroblasts or control transfected Cav1-expressing HS5(+) fibroblasts with or without radiation treatment (10Gy) (B) were removed when tumor volumes reached a critical size (12-22 days after tumor irradiation) and were then subjected to immunohistochemistry with the indicated antibodies. Representative images are shown. Sections were counterstained using hematoxylin. Magnification Cav1, FAP, Tagln 20x; Pcna 10x.

Supplementary Figure S4

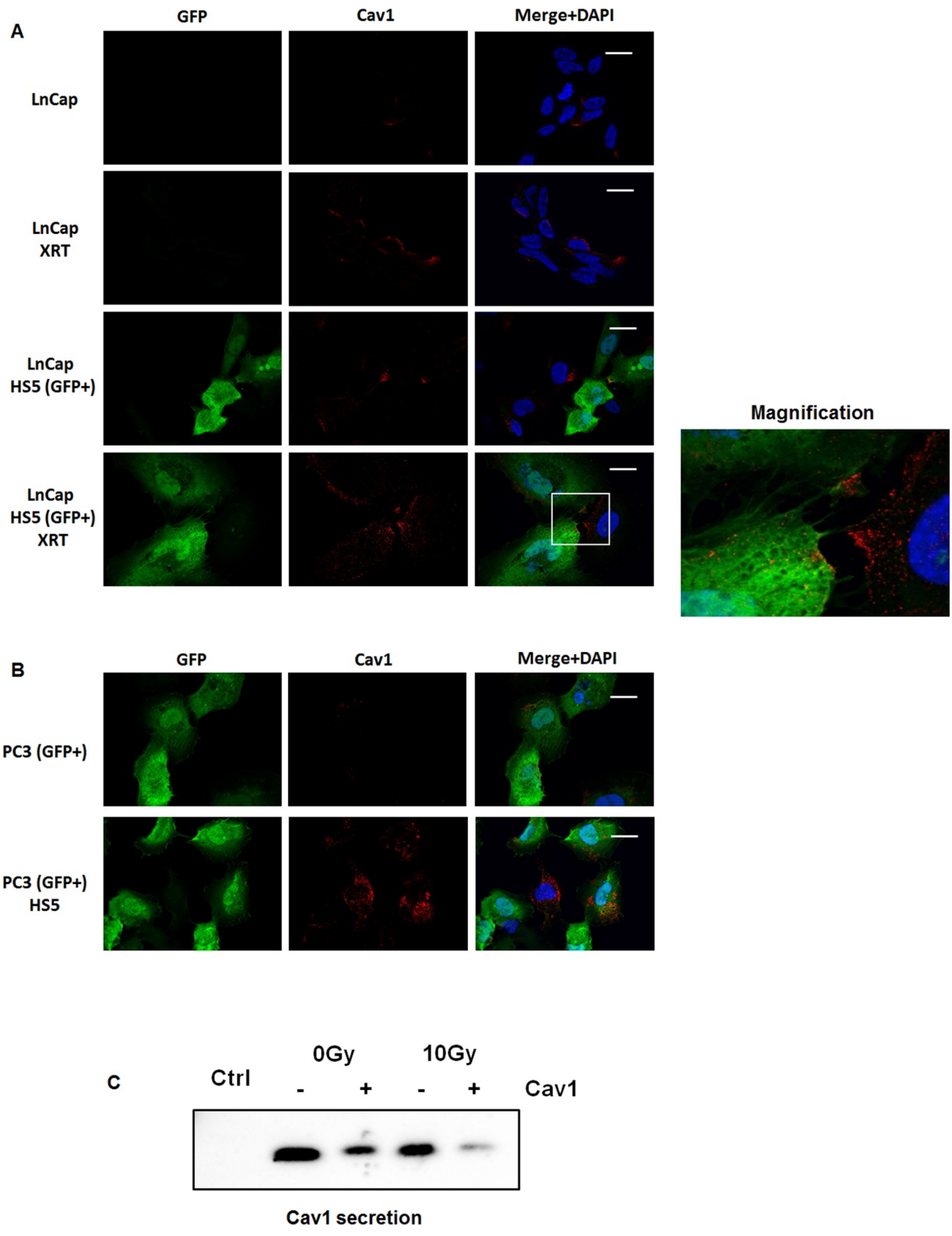

Supplementary Figure S4 | Co-culture of Cav1-expressing HS5 fibroblasts with Cav1-silenced PC3 or Cav-deficient LNCaP malignant epithelial cells yields Cav1-positive cancer cells. (A) Cav1 expression and localization was analyzed in LNCaP cells co-cultured with GFP-expressing (shCtrl)-transfected HS5 fibroblasts by immunofluorescence (red). (B) Cav1 expression was further analyzed in Cav1-silenced and GFP-expressing (shCav1-

transfected) PC3 cell co-cultures with normal (non-transfected**)** HS5 fibroblasts. Nuclei were stained in blue. Representative images from three independent experiments are shown. Magnification 63x (scale bar 25µm). (C) Cav1 secretion was further determined in cell culture supernatants derived from Cav1-silenced HS5(-) or control transfected Cav1-expressing HS5(+) fibroblasts with or without radiation treatment (10Gy) using Western blot analysis. Equal protein amounts (100 µg) were loaded.

Supplementary Figure S5

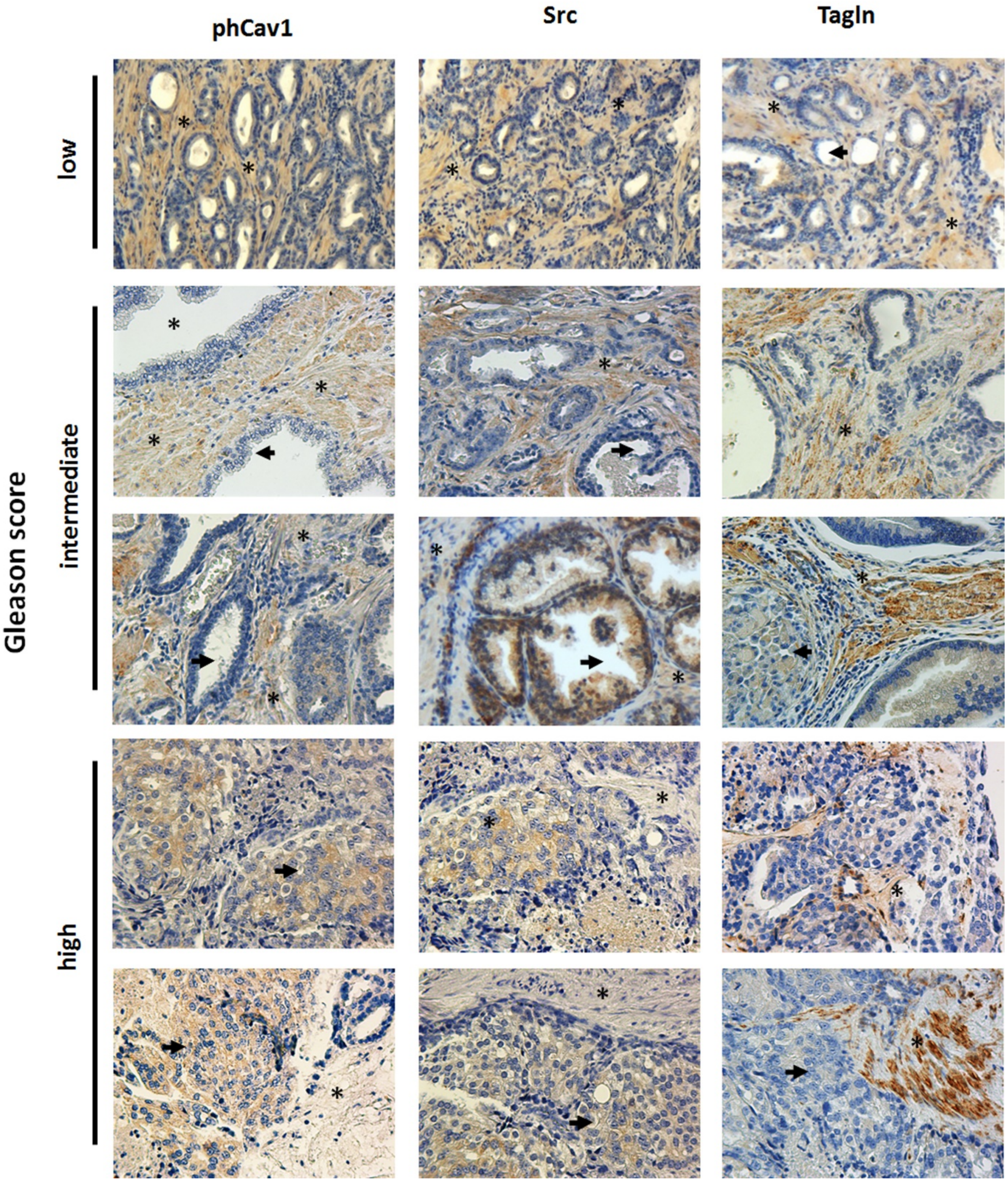

Supplementary Figure S5 | Immunohistological analysis of Cav1 expressions in human prostate tumor tissues (higher magnification photographs). Paraffin-sections of human prostate tumors were stained for the indicated antibodies. Gleason grading scores used to evaluate prognosis of men with prostate cancer were divided into low (1+1, 2+2), intermediate (3+3, 4+3) and high scores (4+5) according to the sum of the primary and secondary Gleason patterns in whole resection specimens. Sections were counterstained using hematoxylin. Representative images are shown. Magnification 40x.

Full Gels

Supplementary Figure S6

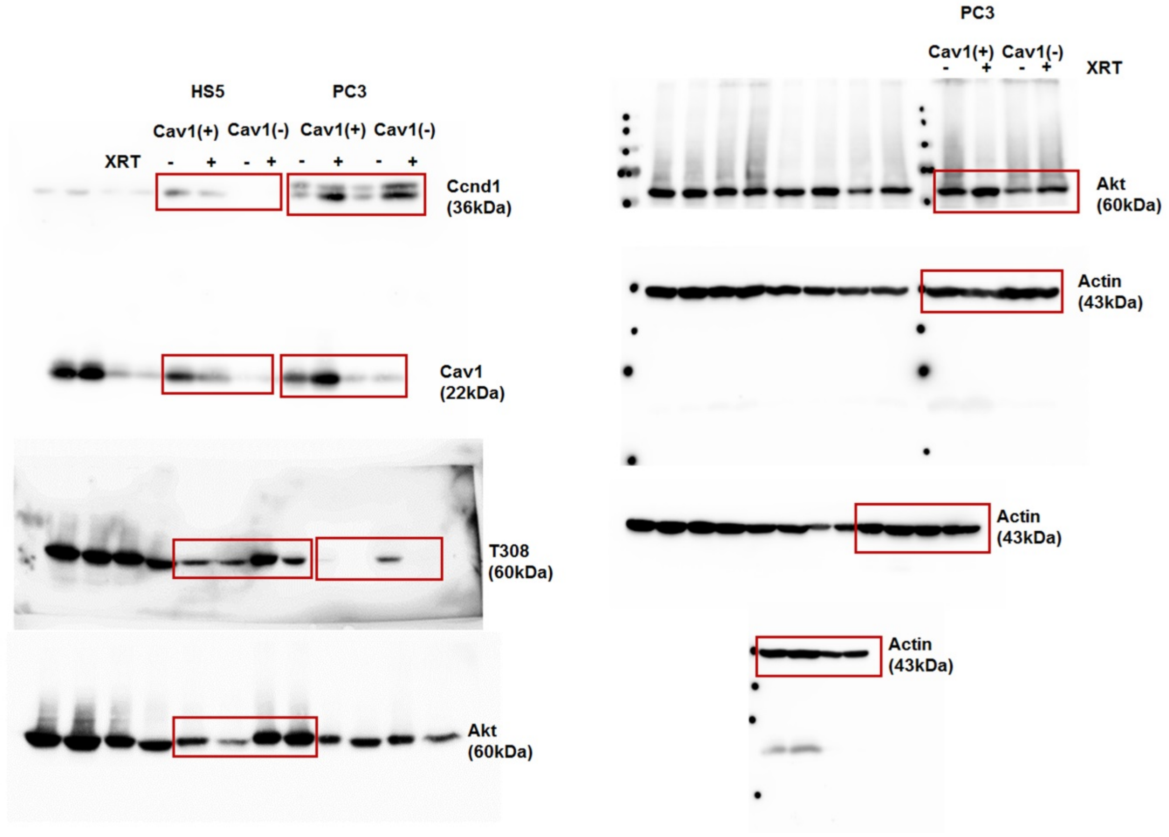

Supplementary Figure S6 | Full gels of cropped gels (emphasized by a red rectangle) as shown in Figure 1C and 5C. Cav1 protein levels were detected by Western blot. Equal protein amounts (50 µg, whole cell lysate) were loaded. Beta-actin was included as a loading control.

Supplementary Figure S7

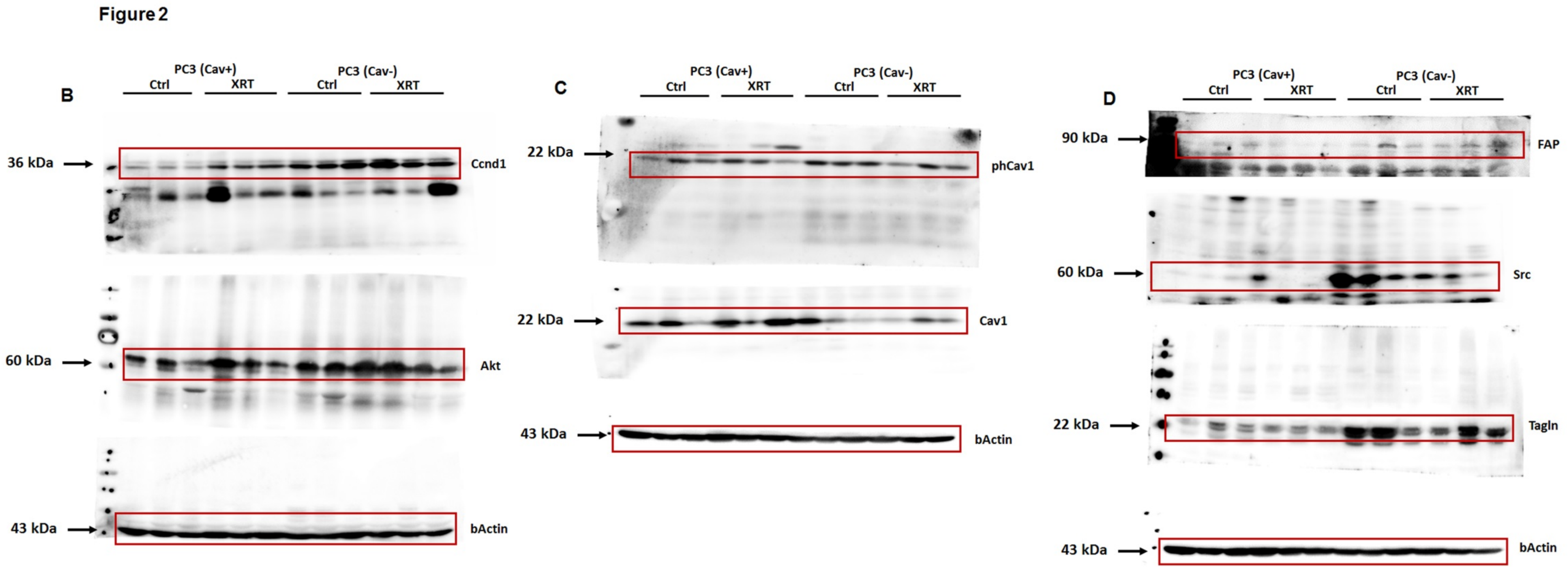

Supplementary Figure S7 | Full gels of cropped gels (emphasized by a red rectangle) as shown in Figure 2B-D. Indicated protein levels were detected by Western blot. Equal protein amounts (50 µg, whole cell lysate) were loaded. Beta-actin was included as a loading control. The Ccnd1 gel (first gel in B) was first probed with a different antibody (~20kDa).

Supplementary Figure S8

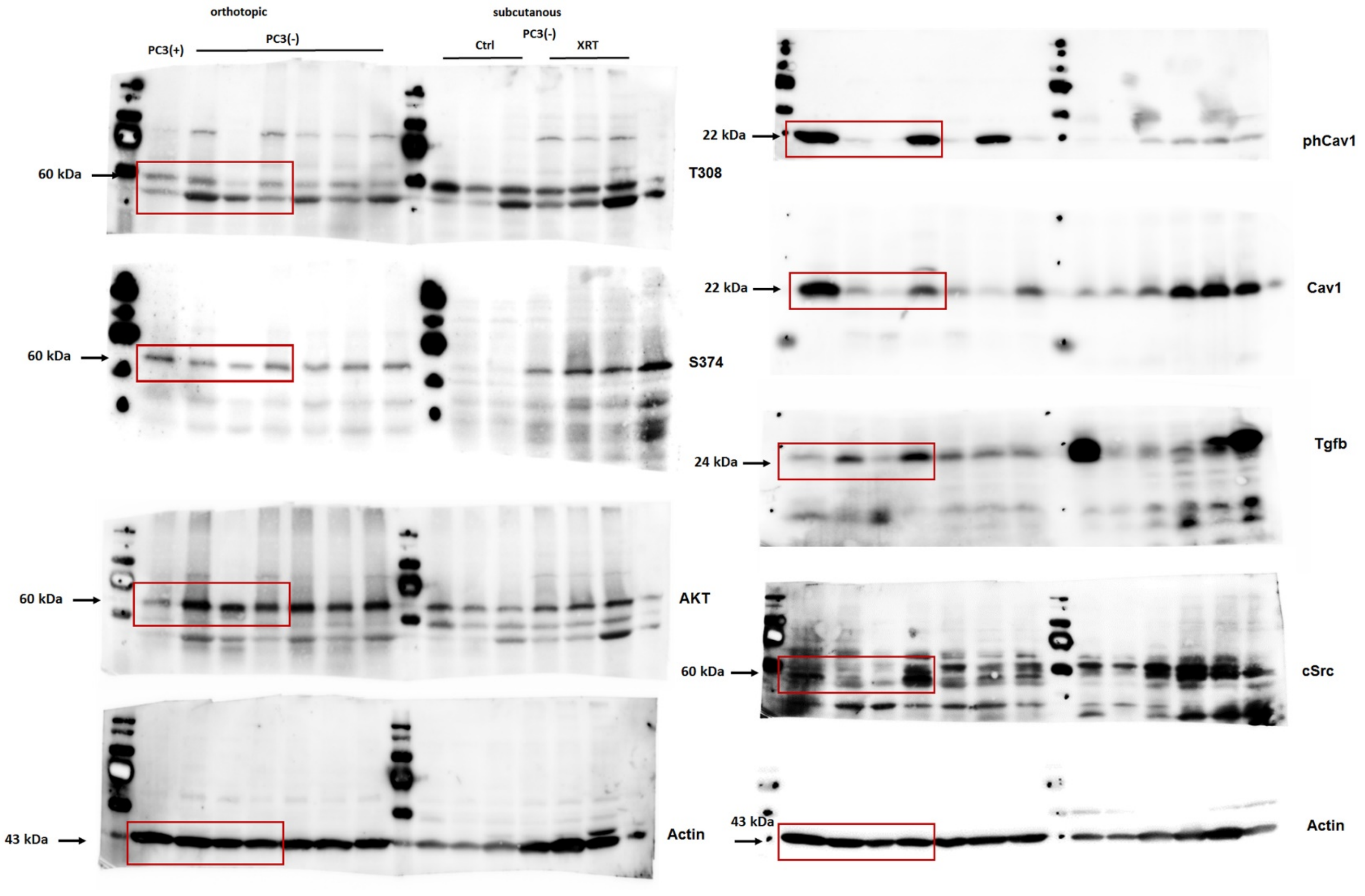

Supplementary Figure S8 | Full gels of cropped gels (emphasized by a red rectangle) as shown in Figure 4B. Indicated protein levels were detected by Western blot. Equal protein amounts (50 µg, whole cell lysate) were loaded. Beta-actin was included as a loading control.

Supplementary Figure S9

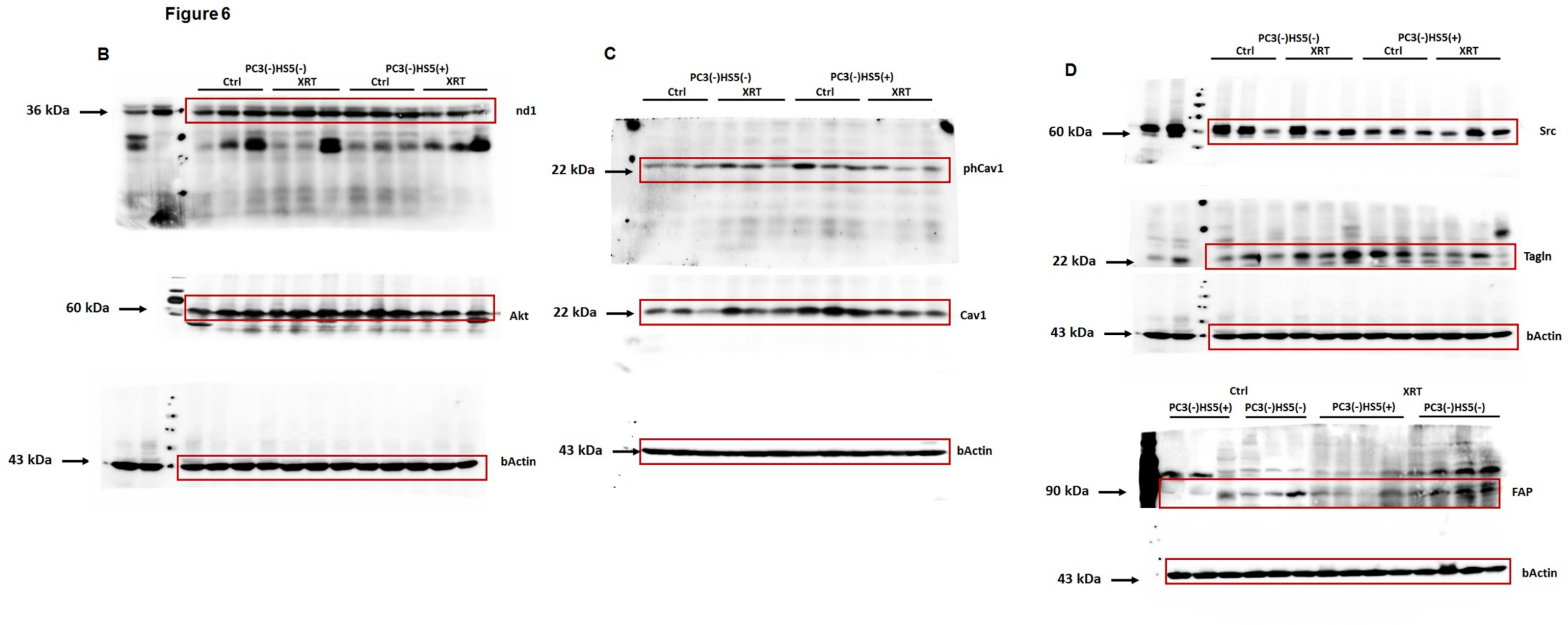

Supplementary Figure S9 | Full gels of cropped gels (emphasized by a red rectangle) as shown in Figure 6B-D. Indicated protein levels were detected by Western blot. Equal protein amounts (50 µg, whole cell lysate) were loaded. Beta-actin was included as a loading control.

Supplementary Figure S10

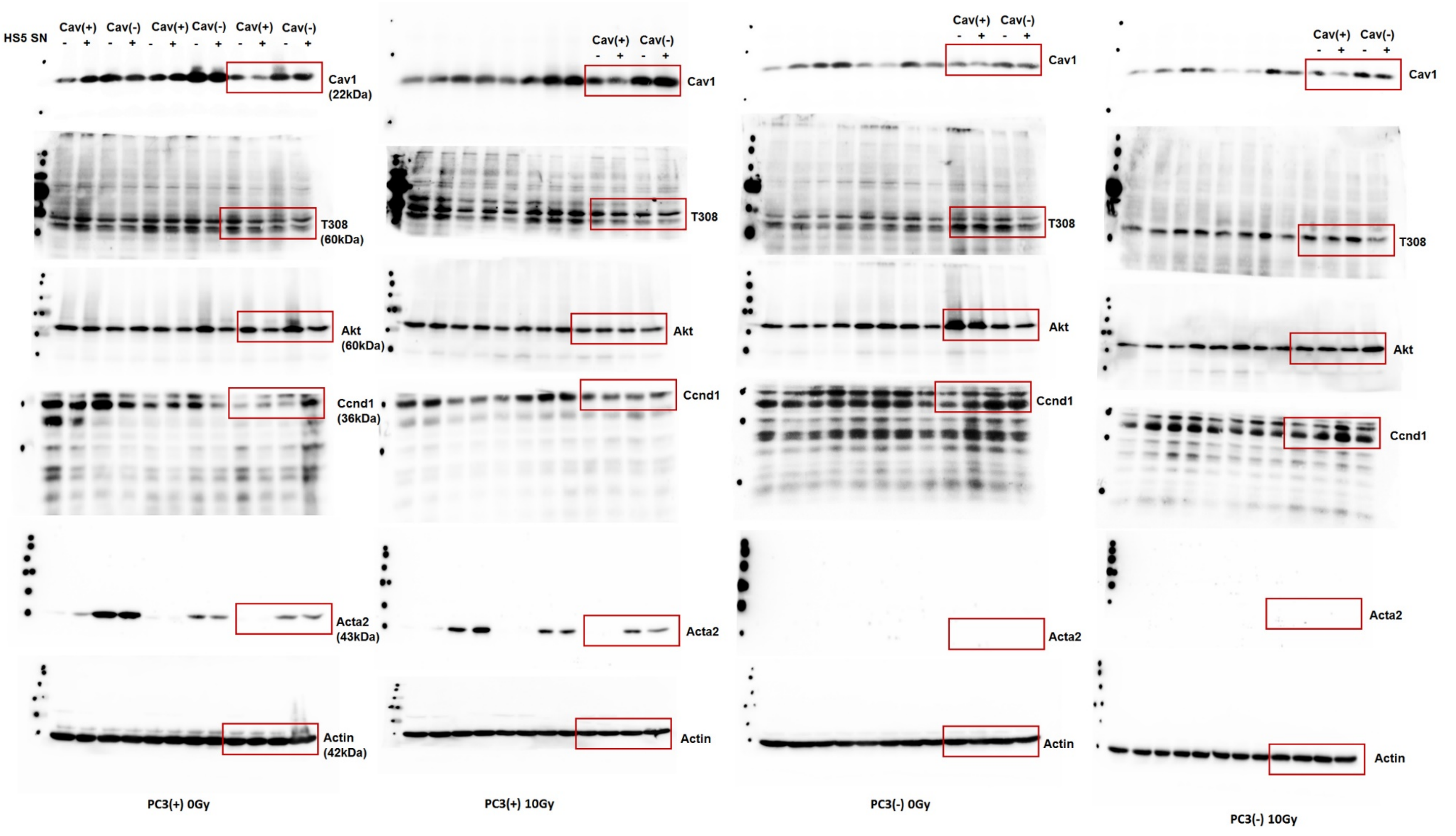

Supplementary Figure S10 | Full gels of cropped gels (emphasized by a red rectangle) as shown in Figure 7A. Indicated protein levels were detected by Western blot. Equal protein amounts (50 µg, whole cell lysate) were loaded. Beta-actin was included as a loading control.
